# Olfactory Epithelium Stimulation Using Rhythmic Nasal Air-Puffs Improves the Cognitive Performance of Individuals with Acute Sleep Deprivation

**DOI:** 10.3390/brainsci14040378

**Published:** 2024-04-13

**Authors:** Hanieh Riazi, Milad Nazari, Mohammad Reza Raoufy, Javad Mirnajafi-Zadeh, Amir Shojaei

**Affiliations:** 1 Department of Physiology, Faculty of Medical Sciences, Tarbiat Modares University, Tehran 14117-13116, Iran; hanieh.riazi@modares.ac.ir (H.R.); raoufy@modares.ac.ir (M.R.R.); mirnajaf@modares.ac.ir (J.M.-Z.); 2 Department of Molecular Biology and Genetics, Aarhus University, 8000 Aarhus, Denmark; mina@dandrite.au.dk; 3 Center for Proteins in Memory—PROMEMO, Danish National Research Foundation, 1057 København, Denmark; 4 Institute for Brain and Cognition, Tarbiat Modares University, Tehran 14117-13116, Iran

**Keywords:** nasal air-puff, non-invasive brain stimulation, acute sleep deprivation, electroencephalography, numerical Stroop test, cognitive function, olfactory epithelium

## Abstract

This study aimed to investigate the effects of intranasal air-puffing on cognitive impairments and brain cortical activity following one night of partial sleep deprivation (PSD) in adults. A total of 26 healthy adults underwent the numerical Stroop test (NST) and electroencephalography (EEG) before and after one night of PSD. Following PSD, subjects in the treatment group (*n* = 13) received nasal air-puffs (5 Hz, 3 min) before beginning the NST and EEG recording. Administration of nasal air-puffs in the treatment group restored the PSD-induced increase in error rate and decrease in reaction time and missing rate in the NST. Intranasal air-puffs recovered the PSD-induced augmentation of delta and theta power and the reduction of beta and gamma power in the EEG, particularly in the frontal lobes. Intranasal air-puffing also almost reversed the PSD-induced decrease in EEG signal complexity. Furthermore, it had a restorative effect on PSD-induced alteration in intra-default mode network functional connectivity in the beta and gamma frequency bands. Rhythmic nasal air-puffing can mitigate acute PSD-induced impairments in cognitive functions. It exerts part of its ameliorating effect by restoring neuronal activity in cortical brain areas involved in cognitive processing.

## 1. Introduction

Sleep is a crucial physiological event that plays a vital role in maintaining overall health and well-being [1]. It is characterized by closed eyes and a profound reduction in responsiveness to sensory stimuli [2]. Sleep constitutes approximately one-third of human life [1]. Having adequate sleep quantity and quality at the appropriate time is crucial for maintaining brain functions, including neuronal regeneration, synapse formation, emotion regulation, and cognitive processes such as learning, memory, attention, and decision-making [2].

The amount of adequate sleep varies among individuals and different age groups, but on average, healthy adults require 7–9 h of sleep each day [3]. Even a slight reduction in sleep duration or quality can lead to sleep deprivation. More than 30 percent of the global population is affected by acute sleep deprivation [4]. Acute sleep deprivation involves a complete lack of sleep or a reduction in sleep duration or quality, typically lasting for one to two days [4]. It results from various factors such as stress, illness, trauma, and environmental conditions. Sleep deprivation can lead to cognitive impairments, changes in mood, sleepiness, and the occurrence of microsleeps [5]. Reasoning abilities, attention, and alertness are among the common cognitive functions that are impaired by sleep deprivation [6]. 

Using EEG recordings, it has been shown that acute sleep deprivation increases delta oscillations in the prefrontal and frontal lobes, augments theta activity in the central, parietal, and occipital regions, and decreases alpha, beta, and gamma rhythms in some brain areas, such as the frontal and parietal lobes [7]. Additionally, acute sleep deprivation reduces inter-hemispheric connectivity and enhances intra-hemispheric brain functional connectivity [7]. Acute sleep deprivation also affects the activity of the default mode network (DMN), which is a brain system exhibiting higher activity during rest [8]. 

Given that sleep deprivation can potentially expose individuals to various health risks, considerable resources have been globally allocated towards enhancing cognitive functions in sleep-deprived individuals. Although methods such as light therapy and meditation have been introduced as strategies to improve cognitive performance in individuals with sleep deprivation, there is clear merit in developing alternative approaches to achieve this objective. Brain stimulation is a method for exciting or inhibiting brain neurons and has shown therapeutic effects in some neurological disorders such as Parkinson’s, epilepsy, and Alzheimer’s disease [9,10]. There is evidence showing that brain stimulation may have a therapeutic effect on improving the cognitive abilities of individuals with acute sleep loss [11]. Treatment of epilepsy patients with invasive deep brain stimulation, in addition to suppressing seizure activity, has been shown to improve the sleep quality and cognitive deficits in these patients [9]. In addition, beyond treating the movement disorder, applying invasive deep brain stimulation is associated with improving sleep quality in patients with Parkinson’s disease [10]. Luber et al. (2013) showed that applying transcranial magnetic stimulation of the brain (at 5 Hz for 7 s) preserved working memory performance during 48 h of sleep loss in human subjects [12]. Moreover, transcranial direct current stimulation (TDCS) over the dorsolateral prefrontal cortex (DLPFC) in the right hemisphere (1 mA for 20 min) increased the functional connectivity of the thalamus with the temporal lobe and improved cognitive performance following 24 h of sleep deprivation [13]. 

Another study demonstrated that a 20-min session of TDCS at 2 mA anodic stimulation over the left DLPFC of healthy military personnel experiencing acute sleep deprivation enhanced cognitive performance, including working memory, attention, and reduced fatigue levels [14,15,16]. Comparable outcomes were observed in medical residents experiencing acute sleep deprivation (sleeping less than 5 h per night) [17]. In another study, exploring the effects of 2 mA TDCS over the DLPFC for 30 min in 30 h sleep-deprived subjects revealed superior cognitive enhancement compared to individuals consuming 200 mg of caffeine [15]. D’Atri et al. (2019) demonstrated that TDCS targeting bilateral frontal-temporal areas at 5 Hz with a maximum current intensity of 0.6 mA for 10 min resulted in heightened attention among participants [18]. Sharma et al. (2018) and Amara et al. (2011) indicated that deep brain stimulation with frequencies exceeding 80 Hz applied to the unilateral or bilateral subthalamic nucleus, medial ventral nucleus of the thalamus, and pedunculopontine nucleus in Parkinson’s patients improved sleep quality and alleviated cognitive symptoms [19,20]. 

However, invasive methods are not suitable for improving cognitive functions in sleep-deprived individuals, and non-invasive methods such as transcranial magnetic stimulation and direct current stimulation have disadvantages such as side effects such as pain, headache, tingling, spasms or twitching of facial muscles [21]. Thus, there is a need to develop a new method for improving the cognitive abilities of sleep-deprived individuals with minimum side effects.

Stimulation of the olfactory epithelium is emerging as another non-invasive method for modulating neuronal activity in brain [22]. The olfactory epithelium comprises olfactory sensory neurons that respond not only to olfactory stimuli but also to mechanical stimulation induced by airflow within the nasal cavity [22]. These neurons project to the olfactory bulb, situated in the ventral anterior region of the forebrain [23]. Neuronal connections originating from the olfactory bulb extend widely throughout the brain, both directly and indirectly affecting neuronal activity in these interconnected regions [22,23]. The influence of respiration on brain dynamics has been increasingly studied in non-olfactory areas over the past decade. 

The breathing rhythm has gained much attention in recent years. Yanovsky and colleagues observed theta and respiration-related oscillations in hippocampal activity under urethane anesthesia during an active state [24]. Studies have confirmed that rhythmic airflow in the nasal nostrils results in respiration-coupled oscillations called respiration-entrained brain rhythm (RR) in the olfactory bulb [25]. Additionally, RR and spiking discharges have been recently observed in other brain areas, including the medial prefrontal cortex (mPFC), orbitofrontal, anterior cingulate, infralimbic, and parietal cortices (for a review see [26]). Notably, these oscillatory activities occur independently of odor perception, as mammalian olfactory sensory neurons, besides containing chemical receptors, possess mechanical receptors and respond to mechanical stimulation by nasal airflow [22]. UP and DOWN occurrences have been found to be influenced by the respiratory phase during slow-wave sleep [27]. Furthermore, respiratory-related entrainment has been observed in various brain areas of patients with refractory epilepsy, including the piriform cortex, amygdala, hippocampus, insula, primary motor and sensory cortices, as well as the frontal regions [25,28]. Additionally, it has been shown that stimulating the nasal mucosa with periodic odorless airflow at a frequency of 0.05 Hz, mimicking an 8 s long inspiration at a constant pressure of 1.1 bar, independent of the subject’s breathing, leads to significant changes in oscillatory activity in the limbic system and DMN structures. This stimulation particularly affects the medial prefrontal cortex and orbitofrontal cortex, resulting in the perception of an altered state of consciousness [29]. Wu and colleagues, using fMRI and local field potential recordings in rats, demonstrated that the activity pattern induced by airflow in the nasal cavity is more widespread than patterns caused by odors [30].

The power of gamma activity, which is essentially involved in cognitive and sensory processing, is modulated by breathing [31]. Modulation of brain oscillations influences information processing and communication between brain regions and plays a crucial role in cognitive performance [32]. Studies have shown that manipulating brain oscillations can enhance cognitive abilities by facilitating neural synchronization and connectivity between brain regions [33]. Based on the above evidence, the aim of the present study was to evaluate the effects of applying rhythmic intranasal air-puffs on cognitive performance and brain activity following acute sleep deprivation. We hypothesized that applying intranasal air puffs can alleviate the cognitive dysfunction of individuals with acute sleep deprivation, as examined by a numerical Stroop test, by restoring brain cortical oscillations and by recovering intra-DMN connectivity. 

## 2. Materials and Methods

### 2.1. Participants

26 volunteers (14 males and 12 females) aged 22 to 36 years old, right-handed, healthy, and without any chronic or acute background diseases, participated in this study. They had normal or corrected vision, reported no substance abuse issues, including drug or alcohol addiction, and were not taking any medications aside from dietary supplements. Five participants were excluded for not following the rules during the experiments. Additional details regarding the demographic information of the participants are provided in Table 1. The ethical considerations of this research were reviewed and approved by the Ethics Committee of Tarbiat Modares University (code: IR.MODARES.REC.1401.085).

### 2.2. Experimental Procedure

**Figure 1:**
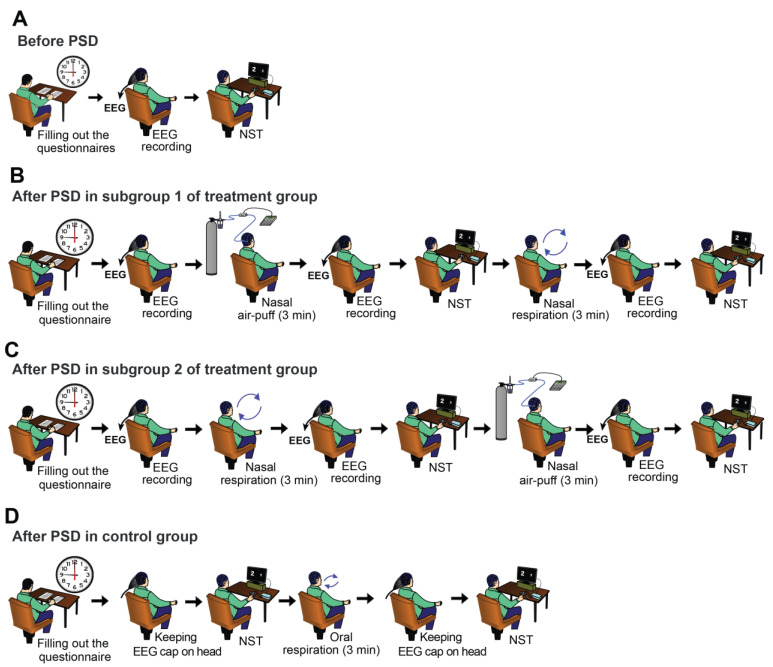
Tasks and experimental design. The order of tasks for the first day (before sleep deprivation) of both control and treatment groups is shown in (**A**). The rest of the figures show the order of tasks on day 2 (after sleep deprivation) for subgroup 1 (**B**) and subgroup 2 (**C**) of the treatment group and for the control group (**D**). PSD: partial sleep deprivation; NST: numerical Stroop test.

The experiments were conducted over two consecutive days, with the entire process spanning approximately 25 h. On the initial day (prior to sleep deprivation), subjects attended the lab at around 9 a.m. In the treatment group, they first completed an informed consent form and then filled out a demographic information table, the Pittsburgh sleep quality index questionnaire, and a cognitive disability questionnaire, respectively. The Pittsburgh sleep quality index questionnaire was used to ensure the inclusion of only individuals without significant sleep disorders in the study. The cognitive disability questionnaire was also used to confirm the absence of severe cognitive impairments in participants. Subsequently, qualified subjects were randomly assigned to control or treatment groups, with the treatment group further divided into two subgroups. Subjects first underwent a 3-min resting EEG recording in a seated position with their eyes open, followed by a 10-min numerical Stroop test (NST), performed according to a method described later (Figure 1A). At the end of the experiment, participants were instructed to abstain from consuming stimulants of the central nervous system such as coffee, tea, energy drinks, or any caffeinated substances for the next 24 h. They were also requested to refrain from napping or engaging in strenuous exercise, with a designated sleep window from 3 to 7 a.m. The following morning (second day), the subjects attended the laboratory at 9 a.m. There was no direct supervision by the experimenter, and participants affirmed their adherence to the experimental protocols by completing a brief questionnaire on the second day (Table S3). 

In subgroup 1 of the treatment group, an EEG recording was initially conducted for 3 min in a resting state. Subsequently, a nasal cannula was inserted into the nostrils, and the rest of the nostril space was obstructed using nasal tampons. Nasal air-puffs were then applied at 5 Hz for 3 min. This was followed by another 3-min resting state EEG recording and the NST. Participants were then instructed to close their mouths and breathe exclusively through their noses for 3 min. Again, a 3-min resting state EEG recording and the NST were carried out (Figure 1B). The procedure for conducting experiments in subgroup 2 was the same as in subgroup 1, except that the timing of nasal air-puffs application and nasal breathing were exchanged (Figure 1C). 

The process of conducting experiments on day two in the control group was as follows: NST was performed at 3 min after normal respiration for 10 min. Afterwards, nasal tampons were inserted into the participants’ nostrils, and they were asked to breathe through their mouths for 3 min. Subsequently, NST was repeated (Figure 1D). EEG recordings were then taken from the subjects in the treatment group. As for the control subjects, an EEG cap with electrodes was placed on their heads for the same duration as the treatment group, but EEG data was not recorded from them.

The results obtained before sleep deprivation on the first day were designated as the non-sleep-deprived state, or “no SD”, and the data acquired after sleep deprivation on the second day were labeled as the sleep deprivation state or “SD”. Accordingly, the results pertaining to the normal breathing state on the second day in the control group were referred to as “SD + Routine resp”, and the results acquired after applying air-puffs or nasal breathing following sleep deprivation on the second day were designated as “SD + Air-Puff” and “SD + Nasal resp”, respectively.

### 2.3. Questionnaires

#### 2.3.1. Pittsburgh Sleep Quality Questionnaire

The Pittsburgh sleep quality index was developed by researchers at the University of Pittsburgh in 1989 [34]. This questionnaire assesses sleep quality by evaluating seven components related to individuals’ sleep over the past month [34]. These components include subjective sleep quality, sleep latency, sleep duration, habitual sleep efficiency, sleep disturbances, use of sleeping medication, and daytime dysfunction [34]. Participants answered the questions on a Likert scale ranging from 0 to 3. The total score from the seven components will range from 0 to 21. This questionnaire has a reliability of 0.83 and has demonstrated high validity in multiple studies. The Persian version of the Pittsburgh sleep quality questionnaire has shown acceptable reliability and validity in the Iranian population [35].

#### 2.3.2. Cognitive Disability Questionnaire

Nejati developed this questionnaire in 2013, which comprises 30 items structured into seven components: memory, cognitive control and selective attention, decision-making, planning, sustained attention, social cognition, and cognitive flexibility [36]. It assesses cognitive disability using a five-point Likert scale ranging from 1 (never) to 5 (always), with higher scores indicating deteriorating cognitive ability. In the Iranian population, the cognitive disability questionnaire has a reliability of 0.83 and has been used in numerous studies [37] (Tables S1 and S2).

### 2.4. Applying Nasal Air-Puffs

**Figure 2:**
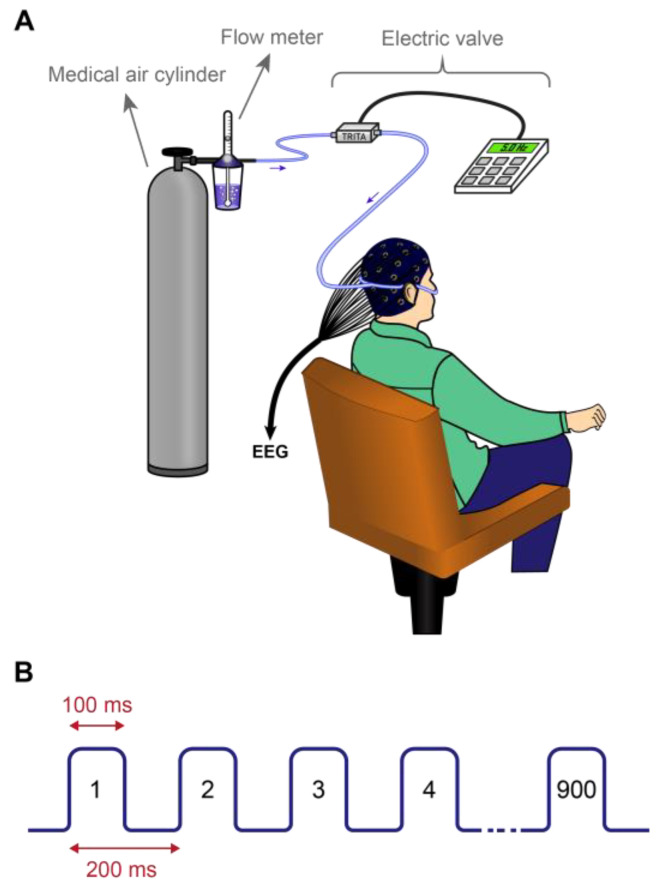
(**A**). a schematic drawing to illustrate the method of applying intranasal air-puffs. (**B**). Pattern of applied intranasal air-puffs. Black numbers denote the pulse number.

Intranasal air-puffs were used to stimulate the olfactory epithelium non-invasively. For this purpose, a nasal cannula was inserted into the nostrils, and the surrounding space was sealed using nasal tampons. The nasal cannula was made of polyhexahydrotriazine and had an inner diameter of 1.5 mm. An electric valve (BIODAC-Ev118, TRITA Health Technology Co., Tehran, Iran), connected to a medical air cylinder, generated rhythmic, odorless, and humidified air-puffs. The frequency of air-puff application was set at 5 Hz (300 puffs per min), with flow rates adjusted to 5.5 or 6 liters per minute. The frequency of intranasal air puffs was adjusted to 5 Hz in order to replicate the frequency of these oscillations, in light of the critical role that theta activities play in cognitive functioning [38]. For every subject, the air-puff flow rate was individually adjusted to the highest level that did not cause annoyance. As a result, participants received nasal air-puffs at 5.5 or 6 L/min. Participants were breathing orally during the administration of the nasal air-puffs (Figure 2).

### 2.5. Numerical Stroop Test (NST)

**Figure 3:**
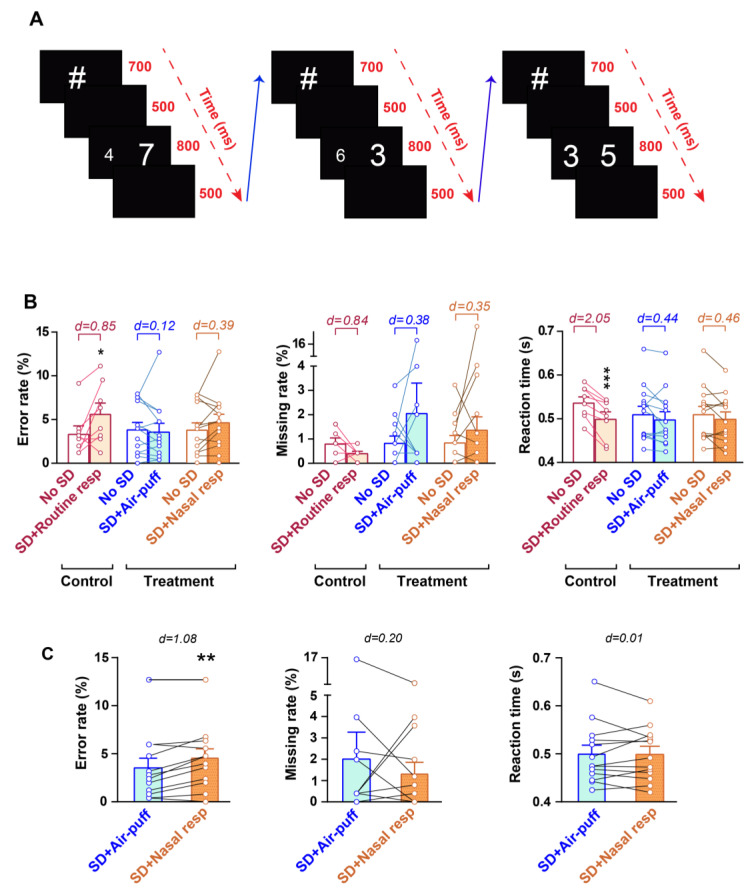
(**A**) The timing protocol for displaying the numbers during the trials of NST. (**B**) Effect of rhythmic nasal air-puff application and nasal respiration on cognitive performance following PSD. (**C**) comparative effects of nasal air-puffing and nasal breathing on subjects’ performance in the NST following PSD. * *p* < 0.05, ** *p* < 0.01 and *** *p* < 0.001; two-way repeated measures ANOVA in (**A**,**B**) and paired *t*-test in (**C**); d shows Cohen’s d number; mean ± SEM.

The NST was used to evaluate cognitive processes as well as the ability to suppress automatic or habitual reactions. Participants in each trial viewed two single-digit numbers on a 16-inch screen situated about 80 centimeters away from their eyes. In each trial, a hashtag symbol was first displayed for 700 ms, followed by a blank screen for 500 ms. Subsequently, a pair of numbers was displayed in white against a black background for 800 ms, which was followed by another 500 ms blank screen. Afterwards, the next trial immediately commenced [39] (Figure 3A). The digits ranged from 1 to 9 and were displayed in the Arial font. Participants were instructed to use a keypad to select the number with the greater value, irrespective of its size, using their right index finger. The side on which the larger number appeared was counterbalanced. The distance between each pair of numbers varied from 1 to 3. The number pairs were presented in three conditions: congruent, where the numerically larger number was physically larger (56 points) than the other digit (32 points); neutral, where both digits were of the same physical size (44 points) but differed numerically; and incongruent, where the numerically larger digit was smaller in physical size. The total number of trials was 252, with an equal distribution across numerical distances and congruency conditions. The trial order was pseudo-randomized. The task was divided into two sessions of 126 trials each, with a 5-min rest period between sessions to minimize mental fatigue and prevent dominating alpha waves.

The following variables were assessed in this test: Error rate: It reflects the percentage of total trials in which the participant provided incorrect responses by selecting a number with a lower value. Reaction time: This parameter represents the average time elapsed between the onset of presenting the numbers and pressing the left or right key. Missing rate: This indicates the percentage of total trials in which the participant failed to press either the left or right key following the presentation of the stimuli. 

The percentage change resulting from PSD in these variables was computed as follows, where applicable:

$$Percentage\;change=\left[\frac{Value\;in\;desired\;state-Baseline\;value}{Baseline\;value}\right]\times 100$$

### 2.6. EEG Recording and Pre-Processing

The participants were comfortably seated in a chair with their eyes open during the recordings. The international 10–20 standard system was used to record EEG data utilizing EEG equipment (V.L430 model, Liv Intelligent Technology, Karaj, Iran) with active electrodes from 31 channels, including Fp1, Fp2, Fz, F3, F4, F7, F8, Cz, C3, C4, T7, T8, Pz, P3, P4, O1, O2, Fpz, CPz, POz, AFz, FC1, FC2, FC6, FC5, CP1, CP2, CP5, CP6, P7, and P8. The right and left mastoids were connected to the ground and reference electrodes, respectively. The data were band-pass filtered between 0.1 and 100 Hz and acquired at 250 Hz. The impedance of the electrodes was kept below 10 kΩ. The recorded signals were imported into the EEGLAB toolbox (version 2023.0; sccn.ucsc.edu/eeglab; accessed on 3 March 2023) in MATLAB for pre-processing [40]. Initially, the signal baseline was adjusted, and the power line artifact was removed by employing a notch filter. Subsequently, the data underwent band-pass filtering between 1 and 100 Hz using a zero-phase linear finite impulse response filter. Additionally, signals at frequencies of 47–53 Hz and 97–100 Hz were band-rejected to remove power line noise. Following artifact rejection through visual inspection, the independent component analysis (ICA) technique was utilized with the “runica” method to eliminate artifacts present in the signal (such as muscles, heart beats, eye blinks, or eye movements). Two to five components were excluded to refine the EEG signal and eliminate unwanted artifacts.

### 2.7. Signal Processing

All signal processing and analysis were conducted in MATLAB (v2022, MathWorks Inc., Torrance, CA, USA). The power of EEG signals was assessed across delta (1–4 Hz), theta (4–8 Hz), alpha (8–13 Hz), beta (13–30 Hz), slow gamma (30–47 Hz), medium gamma (53–70 Hz), and fast gamma (70–97 Hz) frequency bands [41] utilizing the Welch method (4 s sliding humming window with 75% overlap). 

The fractal dimension was calculated to evaluate the complexity of the EEG signals [42]. It is a time domain feature of the EEG that has been demonstrated to effectively distinguish signals from individuals in various activities or conditions [42,43]. Several functions with different algorithms have been developed to estimate the fractal dimension, with two of the most commonly used being Higuchi and Katz.

Higuchi’s algorithm is calculated as follows [44]:

Initially, subsample sets (*X_k_*) are derived from the main sample (*X*) according to the equation:

$$X_{k}^{m}={\left\{X\left(m+ik\right)\right\}}_{i=0}^{\left[\frac{N-m}{k}\right]}$$

where *k* ranges from 1 to *k*_max_, *m* ranges from 1 to *k*, and *N* represents the sample size.

Next, the length of each *X_k_* (denoted as *L_m_*) is computed as:

$$L_{m}\left(k\right)=\frac{\sum_{i=1}^{int(\frac{N-m}{k})}\left|X\left(m+ik\right)-X(m+\left(i-1\right)k\left|\times (n-1)\right.\right.}{k\times int\left[\frac{N-m}{k}\right]}$$

Finally, the fractal dimension (*D*) of the sample is determined based on the relationship:

$$\langle L(k)\rangle \;\propto k^{-D}$$

where 〈*L*〉 signifies the average of *L_m_*. In this study, we analyzed an 80 s duration of the EEG, with *k*_max_ = 8 as specified [45].

The accuracy of Higuchi’s algorithm is higher than Katz’s method, but it is intensely influenced by noise [46]. Subsequently, we also assessed the fractal dimension using Katz’s method [47]. This method involves calculating several parameters: the total sum (*L*) and average (*α*) of the Euclidean distances between consecutive points within the sample, and the maximum distance (*d*) between the initial point and any other point within the sample. The fractal dimension (*D*) of the sample is then determined by the equation:

$$FD=\frac{log(\frac{L}{\alpha })}{\log\left(\frac{d}{\alpha }\right)}=\frac{\log\left(n\right)}{\log\left(n\right)+\log\left(\frac{d}{L}\right)}$$

where *n* represents the ratio of *L* to *α*.

Sample entropy was also calculated to assess the complexity of the EEG signal. It is an improved function of approximate entropy, which was developed by Richman and Moorman (2000) [48]. A higher entropy value indicates a greater number of complex sequences in a series. Sample entropy can be characterized by the space dimension, the standard deviation, and the length of the time series. It is computed as follows:

$$SaEn\;\left(m,r,N\right)=-ln[\frac{A^{m}\left(r\right)}{B^{m}\left(r\right)}]$$

where, *SaEn* (*m*,*r*,*N*) represents sample entropy, *B^m^*(*r*) denotes the estimated probability of two sequences matching for m points, and *A^m^*(*r*) indicates the estimated probability of sequences matching for *m* + 1 points. The values of *A^m^*(*r*) and *B^m^*(*r*) are derived from the data using a relative frequency approach. The embedding dimension should be large enough to capture the underlying dynamics and structure of the EEG signal. The tolerance value determines the level of similarity between data points in the phase space. It should be selected to balance sensitivity to patterns in the data and robustness against noise. In this study, we used standard values for EEG (*m* = 2 and *r* set to 20% of the standard deviation of the 30 s data sequence).

It is noteworthy that Fractal dimension and sample entropy are two different mathematical measures used to assess the complexity of EEG (electroencephalogram) signals. Fractal dimension measures how a signal fills space at different scales. In the context of EEG analysis, fractal dimension quantifies the self-similar or fractal properties of the signal. On the other hand, sample entropy is a measure of signal irregularity or complexity based on the predictability of the signal values. Sample entropy quantifies the likelihood that similar patterns of data points remain similar as additional data points are added to the signal. Both fractal dimension and sample entropy provide valuable insights into the underlying complexity of EEG signals and can be used in combination to gain a more comprehensive understanding of the dynamics of brain activity.

We also evaluated the connectivity between the DMN areas by calculating pairwise coherence and cross-correlation. Coherence was calculated using the MATLAB mscohere function to estimate functional connectivity in the frequency domain, and cross-correlation was computed using the MATLAB xcorr function to evaluate functional connectivity in the time domain.

### 2.8. Statistical Analysis

For statistical analysis, the normal distribution of data was assessed using the Shapiro-Wilk test, and the equality of variances was evaluated using Levene’s test with OriginPro (Version 2022, OriginLab Corporation, Northampton, MA, USA). Statistical comparisons were carried out using GraphPad Prism (version 8, GraphPad Software, Boston, MA, USA). NST parameters between control and treatment groups were compared using a two-way repeated measures ANOVA or an unpaired t-test, where applicable. Within-group comparisons of NST parameters between two states were conducted using the paired t-test. Linear regression was performed to assess the correlation between variables, and the Pearson correlation coefficient was determined to measure the degree of their association. The one-way repeated measures ANOVA with the Tukey post-hoc test was used to compare EEG parameters across different conditions within the treatment group. 

The data are presented as mean ± standard error of the mean (SEM). *p* < 0.05 was regarded as the threshold for determining statistical significance. For multiple comparisons, Tukey’s correction was used when the number of levels of the factors was less than 2, while Bonferroni’s correction was used otherwise.

Cohen’s d was calculated using MATLAB to determine the effect size. An effect size of d < 0.4 was considered weak, 0.4 < d < 0.8 was considered medium, and d > 0.8 was considered strong [49].

## 3. Results

### 3.1. Baseline Characteristics of Participants 

Statistical comparison revealed that there were no significant differences in the age and weight of subjects (*p* = 0.213 for age and *p* = 0.763 for weight; Figure S1A,B) between the control and treatment groups, as well as between subgroup 1 and subgroup 2 of the treatment group (*p* > 0.999 for age and *p* > 0.999 for weight). Furthermore, no significant differences were observed in sleep quality (*p* = 0.680) and cognitive disability indices (*p* = 0.276) between the two groups (Figure S1C,D). Moreover, comparisons of NST parameters, including error rate (*p* = 0.688), missing rate (*p* = 0.938), and reaction time (*p* = 0.304), also did not show significant differences between the two groups on the first day (Figure S2). These results confirm the similar baseline conditions of participants in the control and treatment groups at the beginning of the study.

### 3.2. Cognitive Performance in NST

To investigate the impact of intranasal air-puff application on cognitive performance improvement in NST, the results obtained after sleep deprivation on the second day (referred to as “SD”) in each group were compared with the results obtained before sleep deprivation on the first day (referred to as “no SD”) in the same group. 

Analysis of the NST parameters using a two-way repeated measures ANOVA demonstrated that the effect of time was significant for error rate and reaction time (F_1,31_ = 6.089 and *p* = 0.019 for error rate, F_1,31_ = 0.795 and *p* = 0.379 for missing rate and F_1,31_ = 20.88 and *p* < 0.0001 for reaction time). The treatment effect was not significant for any of the NST parameters (F_2,31_ = 0.193 and *p* = 0.825 for error rate, F_2,31_ = 0.607 and *p* = 0.551 for missing rate and F_2,31_ = 0.192 and *p* = 0.827 for reaction time) but the interaction effect of time × treatment was significant for the error rate (F_2,31_ = 3.421 and *p* = 0.045 for error rate, F_2,31_ = 0.800 and *p* = 0.458 for missing rate and F_2,31_ = 2.850 and *p* = 0.073 for reaction time). The results of the control group indicated that one night of PSD impaired the cognitive performance of participants in NST, as the error rate significantly increased in the SD + Routine resp compared to the no SD state (*p* = 0.016, d = 0.85). Additionally, PSD had a large effect on the decrease in the missing rate (*p* = 0.999, d = 0.84) and a significant effect on reducing the reaction time in subjects of the control group (*p* < 0.001, d = 2.05; Figure 3B and Table S4). 

The application of nasal air-puffs following PSD interestingly restored the cognitive function of subjects in the treatment group, so that none of the NST parameters showed a significant difference between SD + Air-puff and no SD conditions (*p* = 0.999, d = 0.12 for error rate; *p* = 0.399, d = 0.38 for missing rate; *p* = 0.287, d = 0.44 for reaction time; Figure 3B and Table S4). 

We also evaluated the effect of nasal air-puffs following PSD on NST parameters at different numerical distances (i.e., ND1 to ND3) using a two-way repeated measures ANOVA. The results revealed a significant effect of time and the time × treatment interaction in ND1 (F_1,19_ = 12.08 and *p* = 0.002 for time, F_1,19_ = 0.541 and *p* = 0.471 for treatment, and F_1,19_ = 6.259 and *p* = 0.022 for time × treatment), a significant time × treatment interaction in ND2 (F_1,19_ = 0.145 and *p* = 0.707 for time, F_1,19_ = 0.083 and *p* = 0.777 for treatment, and F_1,19_ = 4.531 and *p* = 0.047 for time × treatment) and an insignificant effect of these factors in ND3 (F_1,19_ = 1.028 and *p* = 0.323 for time, F_1,19_ = 0.182 and *p* = 0.675 for treatment, and F_1,19_ = 0.009 and *p* = 0.921 for time × treatment) for the error rate. None of the factors had a significant effect in ND1 (F_1,19_ = 0.006 and *p* = 0.939 for time, F_1,19_ = 0.923 and *p* = 0.349 for treatment, and F_1,19_ = 0.621 and *p* = 0.440 for time × treatment), ND2 (F_1,19_ = 0.497 and *p* = 0.489 for time, F_1,19_ = 1.080 and *p* = 0.312 for treatment, and F_1,19_ = 1.510 and *p* = 0.234 for time × treatment) and ND3 (F_1,19_ = 0.533 and *p* = 0.474 for time, F_1,19_ = 0.843 and *p* = 0.370 for treatment, and F_1,19_ = 0.766 and *p* = 0.392 for time × treatment) for missing rate. However, a significant effect of time and time × treatment was observed in ND1 (F_1,19_ = 19.65 and *p* = 0.0003 for time, F_1,19_ = 0.323 and *p* = 0.576 for treatment, and F_1,19_ = 5.006 and *p* = 0.037 for time × treatment), ND2 (F_1,19_ = 18.62 and *p* < 0.001 for time, F_1,19_ = 0.297 and *p* = 0.592 for treatment, and F_1,19_ = 4.512 and *p* = 0.047 for time × treatment) and ND3 (F_1,19_ = 18.34 and *p* < 0.001 for time, F_1,19_ = 0.294 and *p* = 0.594 for treatment, and F_1,19_ = 4.647 and *p* = 0.044 for time × treatment) for reaction time. Post-hoc analysis revealed that PSD significantly increased the error rate (*p* = 0.002, d = 1.21 between SD + Routine resp and no SD) and had a medium effect on the reduction of missing rate at ND1 and ND2 in control group subjects (*p* = 0.831, d = 0.53 for ND1; *p* = 0.934, d = 0.41 for ND2). In addition, it had a significant effect in shortening the reaction time (*p* < 0.001, d = 2.05 for ND1, *p* < 0.001, d = 2.06 for ND2 and *p* < 0.001, d = 2.00 for ND3 between SD + Routine resp and no SD) at all numerical distances in these individuals (Figure S3 and Table S5). However, nasal air-puffs application recovered the effect of PSD on NST parameters at all numerical distances in the treatment group (*p* = 0.686, d = 0.23 in ND1; *p* = 0.109, d = 0.57 in ND2; *p* = 0.717, d = 0.21 in ND3 for error rate; *p* = 0.817, d = 0.18 in ND1; *p* = 0.250, *p* = 0.35 in ND2; *p* = 0.374, *p* = 0.30 in ND3 for missing rate; *p* = 0.174, d = 0.44 in ND1; *p* = 0.176, *p* = 0.43 in ND2; *p* = 0.192, *p* = 0.42 in ND3 for reaction time; Figure S3 and Table S5). 

As subjects in the treatment group received nasal air-puffs at two different flow rates (i.e., 5.5 and 6 L/min), we aimed to determine whether the effect of nasal air-puffs on cognitive function in NST is influenced by the air-puff flow rate. Statistical analysis revealed no significant difference in any of the NST parameters between the 5.5 and 6 L/min flow rates (*p* = 0.486, d = 0.51 for error rate; *p* = 0.165, d = 0.68 for missing rate; *p* = 0.660, d = 0.27 for reaction time; Figure S4 and Table S6). 

As described in the experimental design, subjects in subgroup 1 of the treatment group experienced nasal air-puffs 23 min before nasal respiration, while subjects in subgroup 2 received nasal air-puffs 20 min after the cessation of nasal respiration. Comparing the NST parameters obtained following the application of nasal air-puffs between the two subgroups of the treatment group did not reveal any significant difference in NST parameters, although a medium effect was observed. This suggests that the effectiveness of nasal air-puffs may be somewhat dependent on the prior respiratory state (usual respiration or nasal respiration) (*p* = 0.458, d = 0.44 for error rate; *p* = 0.315, d = 0.56 for missing rate; *p* = 0.434, d = 0.46 for reaction time; Figure S5 and Table S7). Furthermore, the beneficial impact of nasal air-puffs was not influenced by the subjects’ sleep quality or cognitive disability indices (r = −0.0351, *p* = 0.91 for error rate, r = 0.3568, *p* = 0.23 for reaction time in sleep quality; r = −0.4907, *p* = 0.10 for error rate, r = 0.2588, *p* = 0.42 for reaction time in cognitive disability; Figure S6).

**Figure 4:**
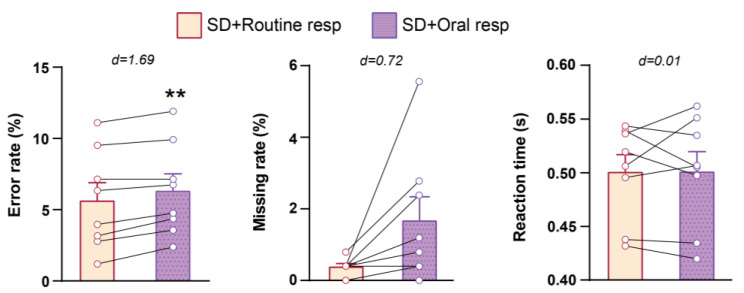
The effect of nasal respiration blockade on subjects’ performance in NST following one night of PSD. ** *p* < 0.01; paired t-test; d shows Cohen’s d number; Mean ± SEM.

Since subjects’ nostrils were occluded by cannulas and tampons during air-puffs application, requiring them to breathe through their mouths, we then investigated whether the closure of the nostrils contributed to the improvement observed with nasal air-puff application. To explore this, we compared the results of NST between the SD + Oral resp (where subjects were breathing through their mouths) and the SD + Routine resp state (where subjects were breathing normally) in the control group following PSD. We found that oral respiration significantly increased the error rate (*p* = 0.002, d = 3.17) and had a medium effect on enhancement of missing rate (*p* = 0.080, d = 0.72). These findings confirm that the stimulation of the olfactory epithelium by air-puff, rather than nostril occlusion, has a beneficial impact on the cognitive function of individuals experiencing acute PSD (Figure 4 and Table S4). 

Investigating the potential positive effect of olfactory epithelium stimulation through nasal respiration on cognitive function following PSD revealed that it had a significant improving effect on the cognitive function of participants following PSD so that it restored the NST parameters to the baseline values of the subjects and only a medium effect was observed in reducing reaction time in the SD + nasal reparation compared to no SD (*p* = 0.630, d = 0.39 in no SD vs. SD + Nasal resp for error rate; *p* > 0.999, d = 0.35 in no SD vs. SD+ Nasal resp for missing rate; *p* = 0.263, d = 0.46 in no SD vs. SD + Nasal resp for reaction time; Figure 3B and Table S4).

We next compared the effect of nasal air-puffs and nasal respiration on the cognitive function of subjects in NST. The result indicated that the application of nasal air-puffs had a greater impact on restoring the cognitive function of participants with acute PSD, as there was a significant difference between the SD + Air-puff and SD + Nasal resp in error rate (*p* = 0.002, d = 1.08; Figure 3C and Table S4). 

### 3.3. EEG Power

**Figure 5:**
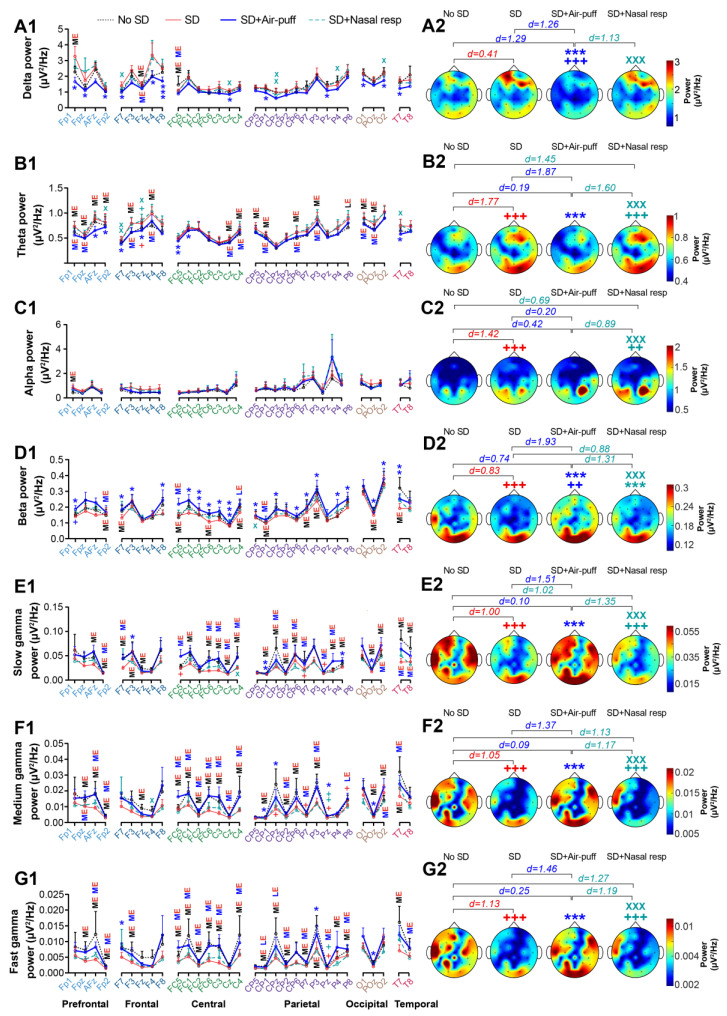
The effect of nasal air-puffs applied following one night of PSD on resting state EEG power. EEG power across various frequency bands for each recording channel is shown in (**A1**–**G1**), and corresponding topographic plots are presented in (**A2**–**G2**). + *p* < 0.05, ++ *p* < 0.01 and +++ *p* < 0.001 compared to no SD; * *p* < 0.05, ** *p* < 0.01 and *** *p* < 0.001 compared to SD; X *p* < 0.05, XX *p* < 0.01 and XXX *p* < 0.001 compared to SD + Air-puff. **ME** indicates the medium effect of SD compared to no SD, **ME** denotes the medium effect of SD + Air-puff compared to SD, **LE** shows the large effect of SD compared to no SD, **LE** indicates the large effect of SD + Air-puff compared to SD; one-way repeated measures ANOVA; d shows Cohen’s d number; mean ± SEM.

To further characterize the effect of nasal air-puffing on cognitive functions following PSD, we also examined its impact on cortical activity in subjects of the treatment group in the resting state using EEG recording. EEG was recorded for 3 min at 4 sessions. The first session was carried out before sleep deprivation (on day 1, referred to as the no SD state), and the rest of the sessions were conducted after PSD (on day 2), consisting of before the application of nasal air-puffs or nasal respiration (referred to as SD), after the application of nasal air-puffs (referred to as SD + Air-puff), and after nasal respiration (referred to as SD + Nasal resp). The results of evaluating EEG power across all recording electrodes are presented on the left side, with corresponding topographical plots derived from the averaged data depicted on the right side of Figure 5. Statistical analysis revealed that PSD enhanced the power of delta and theta oscillations in all cortical lobes, notably in the frontal area. Comparison the topographical plots further confirmed the significant or large effect of PSD on enhancement of delta (*p* = 0.169, d = 0.41) and theta (*p* < 0.001, d = 1.77) power across the entire cranial region. 

Conversely, PSD demonstrated a medium effect on reducing EEG power in the beta and various bands of gamma frequencies, including slow, medium, and fast gamma. Nevertheless, topographical plots illustrating the power of these frequency bands across the skull revealed a significant impact of PSD on decreasing these parameters (*p* < 0.001, d = 0.83 for beta; *p* < 0.001, d = 1.00 for slow gamma; *p* < 0.001, d = 1.05 for medium gamma; *p* < 0.001, d = 1.13 for fast gamma; Figure 5, Tables S8–S14 and S18–S24). It is important to note that we did not observe a noticeable effect of PSD on alpha power across electrodes, except for a medium increase in the Fp1 channel. However, there was a significant difference in the topographical plots of alpha power between the SD and no SD conditions (*p* < 0.001, d = 1.42). 

Interestingly, the application of nasal air-puffs had a restorative effect on the PSD-induced changes in EEG power across various frequencies, including delta, theta and beta, as well as slow, medium, and fast gamma. Comparing the topographical plots confirmed this observation, showing a significant decrease in delta and theta frequencies (*p* < 0.001, d = 1.26 for delta; *p* < 0.001, d = 1.87 for theta) and a significant increase in beta and different gamma frequencies in the SD + Air-puff (*p* < 0.001, d = 1.93 for beta; *p* < 0.001, d = 1.51 for slow gamma; *p* < 0.001, d = 1.37 for medium gamma; *p* < 0.001, d = 1.46 for fast gamma) compared to SD state. The theta and slow, medium, and fast gamma power levels approached baseline values, as indicated by the absence of significant difference between the SD + Air-puff and no SD states (*p* = 0.999, d = 0.19 for theta; *p* = 0.999, d = 0.10 for slow gamma; *p* = 0.999, d = 0.09 for medium gamma; *p* = 0.999, d = 0.25 for fast gamma). In the delta and beta frequencies, nasal air-puffs not only eliminated the PSD-induced changes in EEG power, but also overchanged these parameters, showing a significant difference compared to the no SD state (*p* < 0.001, d = 1.29 for delta; *p* = 0.002, d = 0.74 for beta; Figure 5, Tables S8–S14 and S18–S24). Therefore, the restorative effect of intranasal air-puffing was more prominent at higher frequencies, particularly in the gamma band.

Breathing through the nose for 3 min did not have a significant impact on EEG power across various frequencies. However, upon comparing the topographical plots, a significant increase in beta power was observed following nasal respiration compared to the SD state (*p* < 0.001, d = 0.88). It is worth noting that this observed restorative effect was significantly weaker than that of nasal air-puffs (*p* < 0.001, d = 1.13 for delta; *p* < 0.001, d = 1.60 for theta; *p* < 0.001, d = 1.31 for beta; *p* < 0.001, d = 1.35 for slow gamma; *p* < 0.001, d = 1.17 for medium gamma; *p* < 0.001, d = 1.19 for fast gamma; Figure 5, Tables S8–S14 and S18–S24).

### 3.4. Signals Complexity

**Figure 6:**
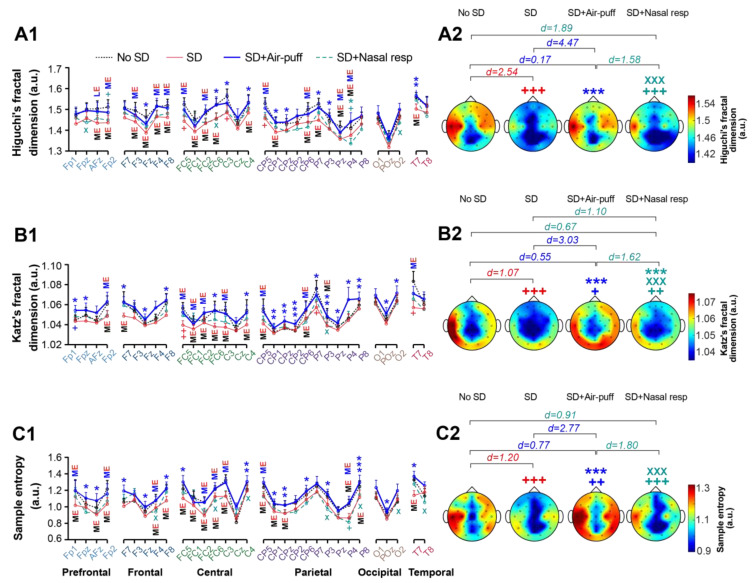
The effect of nasal air-puffs applied following one night of PSD on the complexity of resting state EEG. A comparison of Higuchi’s fractal dimension, Katz’s fractal dimension and sample entropy among different states for each electrode is shown in (**A1**–**C1**), and the corresponding topographic plots are presented in (**A2**–**C2**), respectively. + *p* < 0.05, ++ *p* < 0.01 and +++ *p* < 0.001 compared to no SD; * *p* < 0.05, ** *p* < 0.01 and *** *p* < 0.001 compared to SD; X *p* < 0.05 and XXX *p* < 0.001 compared to SD + Air-puff. **ME** indicates the medium effect of SD compared to no SD, **ME** indicates the medium effect of SD + Air-puff compared to SD, **LE** indicates the large effect of SD + Air-puff compared to SD; one-way repeated measures ANOVA; d shows Cohen’s d number; mean ± SEM.

We then assessed the impact of nasal air-puffs on the complexity of EEG signals recorded during the resting state by calculating the fractal dimension and sample entropy. The analysis of Higuchi’s fractal dimension indicated that acute sleep deprivation had a medium effect on decreasing this parameter in all cortical lobes, except for the occipital cortex, when compared to the no SD state. However, this effect became significant when comparing topographical plots (*p* < 0.001, d = 2.54; Figure 6(A1,A2), Tables S15 and S25). Higuchi’s fractal dimension was restored following the application of nasal air-puffs, showing medium to large and statistically significant effects in increasing this parameter in cortical lobes affected by sleep deprivation in the SD + Air-puff compared to the SD state. This observation was further verified by the significant differences in topographical representations of this parameter between the SD + Air-puff and SD states (*p* < 0.001, d = 4.47). Additionally, a significant difference was noted between nasal air-puffs and nasal respiration, highlighting a stronger restorative effect of nasal air-puffs compared to nasal respiration in recovering the fractal dimension (*p* < 0.001, d = 1.58; Figure 6(A1,A2), Tables S15 and S25).

As Higuchi’s algorithm for determining the fractal dimension is highly sensitive to noise, we also calculated this parameter using Katz’s method. The results affirmed the observations made with Higuchi’s method, indicating a significant decrease in the topographic plots of the fractal dimension due to acute PSD (*p* < 0.001; d = 1.07 in SD compared to the no SD state), which was significantly restored following the application of nasal air-puffs (*p* < 0.001; d = 3.03 between SD + Air-puff and SD). Moreover, the efficacy of nasal air-puffs in restoring Katz’s fractal dimension was significantly more than nasal respiration (*p* < 0.001; d = 1.62 between SD + Nasal resp and SD + Air-puff; Figure 6(B1,B2), Tables S16 and S26). 

Calculating the sample entropy to determine EEG complexity, revealed a significant reduction following acute PSD (*p* < 0.001; d = 1.20 between SD and no SD states) across cortical regions in topographic plots. The application of nasal air-puffs restored this parameter, showing a significant difference in the SD + Air-puff condition compared to SD state (*p* < 0.001, d = 2.77). Once again, this effect was notably superior to nasal respiration (*p* < 0.001, d = 1.80 between SD + Nasal resp and SD + Air-puff; Figure 6(C1,C2), Tables S17 and S27).

### 3.5. Intra-DMN Functional Connectivity

#### 3.5.1. Cross-Correlation

**Figure 7:**
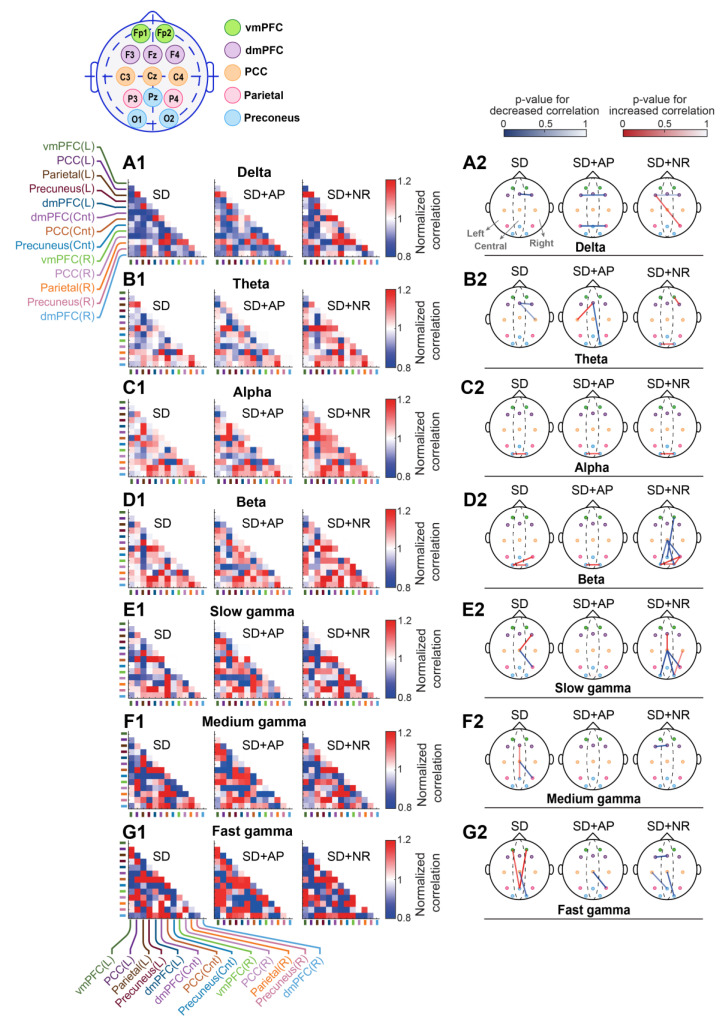
The effect of applying nasal air-puffs subsequent to PSD on cross-correlation within DMN during resting state. The inset shows the electrodes corresponding to DMN areas. (**A1**–**G1**) display the cross-correlation of each state across various frequency bands normalized to the no SD. Each cell represents the normalized cross-correlation between thirteen electrodes for DMN. The location of the recording channels on the skull is mentioned as L (left), Cnt (center) and R (right). (**A2**–**G2**) depict *p*-values indicating statistical differences in pairwise cross-correlation of DMN areas between each state and the no SD condition across different frequency bands. Blue colors reflect decrease and red colors represent increase in coherence. vmPFC: ventromedial prefrontal cortex, dmPFC: dorsomedial prefrontal cortex, PCC: posterior cingulate cortex, SD: sleep deprivation, SD + AP: sleep deprivation + nasal air-puffs, SD + NR: sleep deprivation + nasal respiration; paired *t*-test.

As EEG was recorded during the resting state in this study, we next assessed the functional connectivity between DMN regions exhibiting the highest activity during resting state. Functional connectivity was examined across various frequency bands in both the time and frequency domains by computing cross-correlation and coherence, respectively. The electrodes corresponding to each DMN area are shown in the inset of Figure 7.

Figure 7, Table S28 depicts matrices illustrating the pairwise cross-correlation of DMN areas on the left and the *p*-values representing changes in cross-correlation between paired areas on the right. The *p*-values denote the statistical comparison between each state and no SD. The weak changes are removed from the skull plots. As observed in this figure, one night of PSD altered the intra-DMN network correlations. Notable alterations included a decreased correlation between central and right portions of dmPFC in delta, decreased correlation between the central and right areas of the dmPFC, and between the central dmPFC and the right PCC in the theta band. Additionally, there was an increased correlation between the left and right precuneus in the alpha band, an enhanced correlation between the left precuneus and the right parietal cortex, and between the left and right portions of the precuneus in the beta band. Moreover, there was a decline in correlation between the central PCC and the right parietal cortex, and a heightened correlation between the central PCC and the right dmPFC in the slow gamma band, a reduced correlation between the central PCC and the right parietal cortex, accompanied by an augmented correlation between the central dmPFC and the central precuneus in the medium gamma band. Furthermore, there was a decreased correlation between the central PCC and the right precuneus, along with an enhancement in correlation between the central precuneus and the left and right portions of the vmPFC in the fast gamma frequencies. 

The application of nasal air-puffs not only did not fully amend the PSD-induced alteration in intra-DMN correlation at low frequencies (i.e., delta and theta), but it also led to more reduction in this parameter. It involved diminished correlation between the left and the right dmPFC and between the left and right parietal cortex in the delta band, enhanced correlation between the central dmPFC and the left PCC, and reduced correlation between the central dmPFC and the right precuneus in the theta band. No considerable effect on cross-correlation was observed at alpha frequencies. However, the improvement effect of nasal air-puffs was noticeable at higher frequencies (beta and gamma) as it largely eliminated the changes induced by PSD in DMN connectivity. only an augmented correlation between the left and right precuneus in the beta band and a decreased correlation between the central PCC and the right parietal cortex in fast gamma frequencies were observed compared to the no SD state. 

Nasal respiration not only failed to remove the PSD-induced changes in intra-DMN connectivity, but it also caused additional changes in various frequency bands, suggesting that it had a less restorative effect, especially at beta and gamma frequencies compared to nasal air-puffs.

#### 3.5.2. Coherence

**Figure 8:**
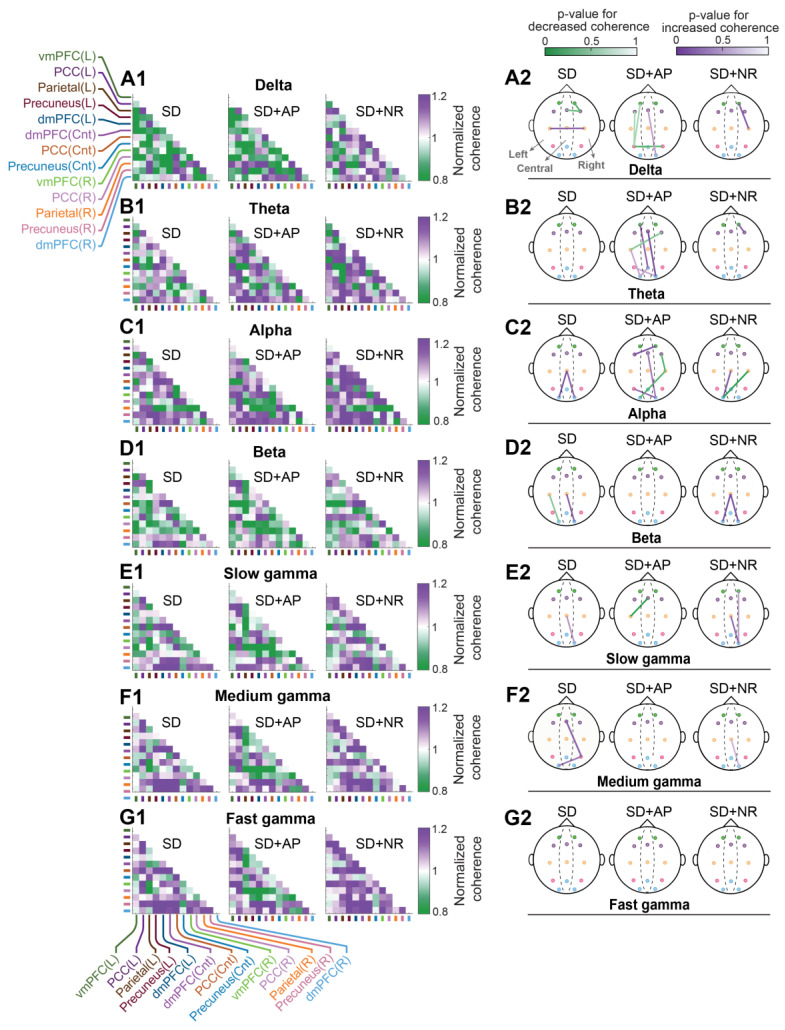
The effect of nasal air-puffs applied following PSD on coherence within DMN during resting state. (**A1**–**G1**) display the coherence of each state across various frequency bands normalized to the no SD. Each cell represents the normalized coherence between thirteen electrodes for DMN. The location of the recording channels on the skull is mentioned as L (left), Cnt (center), and R (right). (**A2**–**G2**) depict *p*-values indicating statistical differences in pairwise coherence of DMN areas between each state and the no SD condition across different frequency bands. Green colors reflect decrease and purple colors represent increase in coherence. vmPFC: ventromedial prefrontal cortex, dmPFC: dorsomedial prefrontal cortex, PCC: posterior cingulate cortex, SD: sleep deprivation, SD + AP: sleep deprivation + nasal air-puffs, SD + NR: sleep deprivation + nasal respiration; paired *t*-test.

Analysis of coherence revealed that one night of PSD induced changes in intra-DMN connectivity across various frequency ranges (Figure 8, Table S29). Notably, a decrease in coherence was observed between the right dmPFC and the right vmPFC, as well as the central dmPFC, accompanied by an increase between the right and left PCC in the delta band. Additionally, coherence displayed an increase between the central PCC and the left and right precuneus. Furthermore, a decrease in this parameter was noted between the left PCC and the left precuneus, along with an increase between the central PCC and the right precuneus in the beta frequencies. Moreover, an increase in coherence was observed between the central PCC and the right precuneus in the slow gamma band, and between the central dmPFC and the right parietal cortex, as well as between the right parietal cortex and the left precuneus in the medium gamma frequencies. Theta and fast gamma frequencies remained unaffected by PSD.

Similar to the findings on cross-correlation, the application of nasal air-puffs had a restorative effect on intra-DMN connectivity, especially at high frequencies comprising beta and gamma bands, as it eliminated PSD-induced changes in DMN connectivity (Figure 8, Table S29). Although there was a reduced coherence between the central dmPFC and the left PCC in the slow gamma band. However, in delta and theta frequencies, the application of nasal air-puffs following PSD led to more alterations in DMN connectivity, including reduced coherence between the left parietal cortex and the left vmPFC, left dmPFC, central precuneus, and right parietal cortex. It also resulted in an increase in coherence between the central dmPFC and the right precuneus in the delta band. Additionally, it caused a decrease in coherence between the right dmPFC and the left PCC, as well as an increase in coherence between the right precuneus and the central dmPFC and the left parietal cortex, between the central precuneus and the left vmPFC and the left precuneus, and between the left precuneus and the left PCC in the theta band.

Compared to nasal air-puffs, nasal breathing had a greater restorative effect on intra-DMN connectivity following one night of PSD in delta and theta frequencies. However, nasal breathing did not show a significant improvement in DMN coherence in the alpha, beta, and slow gamma bands. Nevertheless, it restored intra-DMN connectivity in the medium and fast gamma bands.

## 4. Discussion

The results of this study indicated that administration of a 3-min nasal air-puff following one night of PSD ameliorated cognitive function impairment observed in the NST. Additionally, this intervention significantly reversed the PSD-induced changes in the distribution of high-frequency EEG power and complexity across the scalp, along with alterations in intra-DMN functional connectivity within the beta and gamma frequencies. 

Numerous studies have consistently demonstrated a decline in cognitive functions following periods of sleep deprivation. Working memory, episodic memory, and associative memory are negatively affected by sleep deprivation [50,51]. Furthermore, increased fatigue resulting from sleep deprivation impairs alertness and attention [50]. Moreover, sleep deprivation extends its influence to various cognitive domains, including logical reasoning, social judgment and decision-making [51,52]. In the present study, one night of PSD impaired the cognitive function of subjects, as indicated by an elevated error rate in the NST. Specifically, the heightened error rate was observed at trials with a numerical distance of 1 (which is considered the most challenging trials among the numerical distances), indicating a decrease in the cognitive capacity of the subjects following PSD. The performance of subjects in this test relies on various aspects of brain function, such as attention, logical thinking, and mathematical reasoning [53]. Therefore, the correct response in this test necessitates the interaction of diverse brain regions [53].

Previous studies have shown that after acute sleep deprivation, attention becomes highly unstable and irregular, leading to variable performance in attention-related tests [50]. However, overall individuals’ performance in attention tests decreases following sleep deprivation as a result of increased sleep pressure and decreased alertness [50,51]. Generally, the overall duration of wakefulness during the sleep deprivation period can predict the extent of attention disruption [50].

In the present study, the EEG evaluation subsequent to PSD validated the prior findings and demonstrated an elevation in power of low-frequency oscillations (delta and theta) and reduction in power of high-frequency bands (beta and gamma) across diverse cortical regions, notably in the prefrontal and frontal areas. Consistent with these findings, analysis of EEG signals also revealed a decline in signal complexity, particularly in frontal areas, likely due to augmented power in low-frequency bands which have higher amplitudes and diminished power in high-frequency bands which have lower amplitudes. These changes indicate increased synchrony of neuronal activity across the cerebral cortex following sleep deprivation, which may result in a decline in their processing capacity. Given the pivotal role of frontal brain regions in higher cognitive processes such as reasoning, problem-solving, attention, and decision-making, these changes can, to some extent, explain the impaired cognitive function following sleep deprivation [54,55]. However, we found a decrease in delta power in FC5 and an increase in beta and gamma power in FC5, Fp2 and O2 electrodes following PSD. These alterations likely indicate compensatory increases in neural activity in the relevant areas in response to sleep deprivation, somehow allowing the brain to preserve cognitive functions. Furthermore, no significant alterations in the alpha frequency band in EEG were observed following PSD. It has been proposed that alpha oscillation power increases during a relaxed mental state [56]. Perhaps due to the assessment of EEG during the resting state in this study, no significant differences or large effects in alpha frequencies were noted following PSD.

Studies using EEG recordings and fMRI have shown that one night of sleep deprivation alters the functional connectivity of various brain areas, including a decrease in brain networks integration [57]. As a result, discrimination between brain networks is reduced in fMRI by sleep deprivation, causing a reduction in vigilance and impaired cognitive functions [57,58,59]. 

Interestingly, our study showed that one night of acute PSD decreased reaction time and the missing rate in the NST. Although these results may seem unexpected at first, they can be justified according to previous findings. In a fMRI study, Venkatraman et al. (2007) reported that the right nucleus accumbens was more activated following taking the riskier choices in a gambling task following sleep deprivation compared to a normal sleep condition, indicating an elevated expectation of the higher reward after taking the decision [60,61]. In addition, the insular and orbitofrontal cortices were less activated following losses, indicating a reduced response to losses [61]. Olson et al. (2016) and Killgore et al. (2006) observed that sleep-deprived individuals performing the Iowa Gambling Task exhibit impulsive behaviors, making riskier decisions more easily and considering short-term rather than long-term benefits [62,63]. In another study, Mullin et al. (2013) observed heightened activity in the ventral striatum and decreased deactivation in the mPFC during the winning in a monetary reward task following a night of sleep deprivation compared to a non-sleep-deprived state [61]. Together, these findings suggest that the value of reward and punishment is not accurately represented in neuronal activities following sleep deprivation, leading to impulsive and erroneous decisions [50]. This may explain the decreased missing rate and reaction time despite the increased error rate observed in control subjects following sleep deprivation.

Administration of intranasal air-puffs improved the cognitive performance of participants following a night of PSD in this study, as evidenced by the recovering the error rate, missing rate, and reaction time to baseline values. The increase in error rate during more challenging trials was notably mitigated when nasal air-puffing was administered. These findings indicate an improvement in cognitive abilities in sleep-deprived individuals that was independent of their pre-existing sleep quality and cognitive disability. 

The present study constitutes the first investigation into the use of intranasal air-puffs for non-invasive brain stimulation to improve cognitive abilities in sleep-deprived individuals. The observed ameliorating effects of intranasal air-puffs on cognitive performance are most likely attributable to the stimulation of the olfactory epithelium, as blockade of nasal nostrils impaired the cognitive performance of subjects in the control group. Interestingly, intranasal air-puffs exhibited greater efficacy in ameliorating cognitive functions compared to nasal respiration following PSD, suggesting a potential dependency on stimulation frequency (and possibly the duration of each air-puff pulse). However, more studies are required to further elucidate this hypothesis. The olfactory bulb, serving as the initial relay station in the olfactory pathway, receives input from olfactory sensory neurons in the olfactory epithelium and sends projections to key brain regions implicated in cognition, such as the piriform cortex and orbitofrontal cortex [64]. These regions are involved in processing olfactory information and forming associations with memory, emotions, and decision-making [64]. Furthermore, olfactory stimulation has been demonstrated to activate brain areas associated with memory consolidation, including the hippocampus, suggesting a role for the olfactory system in memory encoding and retrieval [65]. Moreover, olfactory cues have been linked to emotional processing in regions such as the amygdala, impacting cognitive functions related to mood and behavior [66]. Studies have also revealed the relay of olfactory information to the prefrontal cortex, where it may modulate attentional processes [67]. The orbitofrontal cortex, which is involved in olfactory processing, contributes to attentional control and decision-making [67]. Additionally, the amygdala, a key player in emotional regulation, maintains connections with both the olfactory system and attention networks [66]. Furthermore, the ACC, recognized for its role in cognitive control, likely plays a role in integrating olfactory input with attentional mechanisms [68].

As the olfactory bulb has extensive direct or indirect connections with different brain regions, activating the olfactory epithelium and subsequent activation of the olfactory bulb can influence activity in various brain regions [69]. In a seminal study, Grosmaitre et al. (2007) demonstrated that sensory neurons in the mammalian olfactory epithelium exhibit mechanosensitivity [22]. They showed that these neurons not only respond to chemical stimuli, but they are also activated by mechanical stimuli [22]. Grosmaitre et al. (2007) proposed that mechanical stimuli employ an intracellular signaling pathway similar to that used by olfactory stimuli to activate olfactory sensory neurons [22]. Apart from odorants, a few studies have demonstrated that olfactory epithelium stimulation by rhythmic intranasal air-puffs has therapeutic potency for ameliorating impaired cognitive function in abnormal brain states [29]. In one study, Ghazvineh et al. (2021) reported enhanced oscillatory activity across brain regions playing a key role in cognitive functions and learning and memory by applying rhythmic air-puffs into the nasal cavity of animals under mechanical ventilation [70]. This effect was accompanied by improved working memory in these animals [70]. In another study, Salimi et al. (2022) found that nasal air-puffing enhanced the power of gamma oscillations in the DMN of comatose patients’ brains [45]. It also heightened coherence and synchrony between DMN regions [45]. 

In parallel to these findings, stimulation of the olfactory epithelium with nasal air-puffing in the current study interestingly had a recovering effect on PSD-induced changes in cortical activity by diminishing the power of delta and theta, and augmenting the power of beta and gamma bands in various cortical regions. Additionally, it increased the signal complexity in most brain lobes, particularly in the prefrontal and frontal lobes, which have a key role in higher cognitive functions. 

Although cortical delta and theta oscillations have been implicated in cognitive processes, previous studies have predominantly focused on elucidating the role of high-frequency oscillations (beta and gamma) in cognitive functions [71]. Beta waves in the frontal lobe are linked to attention, working memory, decision-making, and motor planning [72]. In the parietal lobe, they play a role in motor-sensory integration, spatial awareness, and motor control, while in the temporal lobe, they are involved in auditory information processing, language comprehension, and memory formation [72]. Occipital lobe beta waves contribute to visual perception and attention [72]. Gamma waves are also essential for cognitive processes, facilitate information processing and integration, particularly in the prefrontal cortex [73]. In the parietal lobe, they assist in sensory integration, spatial perception, and attention, linking sensory information with external world representation [73]. Gamma waves in the temporal and occipital lobes are associated with sensory processing, perception, and memory [73]. 

It is noteworthy that the administration of nasal air-puffs in this study could not restore the DMN connectivity in the delta and theta bands, demonstrating that it does not mitigate all PSD effects on cortical activity. However, nasal air-puffing noticeably restored the intra-DMN functional connectivity at beta and gamma frequencies. Consistent with this finding, it has been reported that there is a cross-area modulation of gamma activity in the primary somatosensory cortex, primary motor cortex, and primary visual cortex phase-locked to the rat’s slow respiratory rhythm (approximately 2.5 Hz) at rest [74]. The DMN appears to play a crucial role in cognitive functioning [75]. Functional and structural disruptions of the DMN have been observed in individuals with amnestic mild cognitive impairment [76]. Furthermore, abnormal functional connectivity within the DMN during inter-ictal periods in childhood absence epilepsy has been documented, indicating atypical anatomo-functional integration within the DMN, potentially contributing to cognitive dysfunction and unconsciousness during absence seizures [77]. In contrast, increased DMN connectivity may be linked to fundamental aspects of mindfulness and self-cognitive skills [78]. 

Therefore, the application of air-puffs in our study was able to recover the cognitive performance of participants following sleep deprivation, in part by restoring the high frequency oscillations in neuronal activity of the cortex that play important roles in cognitive processes. In support of this hypothesis, it has been shown that olfactory epithelium stimulation increases gamma oscillations in the brain [79]. It also influences emotional processing and memory formation [80]. Additionally, Qun Li et al. (2023) showed that closed loop electrical stimulation of the olfactory bulb facilitated the transmission of gamma oscillations from the olfactory bulb to limbic structures and led to a reduction in depressive-like behaviors [81]. This stimulation also modulated the gamma oscillations in cortical regions [81]. 

One of the most important brain nuclei whose activity is influenced by respiration is the amygdala [25]. This area receives direct projections from the OB and/or piriform cortex (for a review, see [82]). Importantly, mPFC and orbitofrontal cortex are densely connected with amygdala [83]. Therefore, the amygdala may be involved in transmitting respiratory signals from the olfactory bulb to higher brain structures [26].

The precise mechanisms responsible for the effects of olfactory epithelium stimulation on cognitive functions and modulation of brain rhythms remain largely unexplored, and more studies are required to elucidate these mechanisms.

The current study had a few limitations, including the absence of a sham stimulation condition to control for potential placebo effects and the relatively small sample size of the study, which may limit the generalizability of the conclusions. In addition, this study does not investigate the impact of intranasal air-puff in cases of acute complete sleep deprivation and chronic sleep deprivation, potentially limiting the generalizability of the findings to these particular populations.

The findings of the study indicate that intranasal air-puff may have clinical implications as a therapeutic intervention for improving cognitive function in individuals experiencing acute sleep deprivation. This treatment modality could potentially aid in reducing the incidence of road traffic accidents among sleep-deprived drivers. Future research should explore the development of non-invasive devices that can stimulate the olfactory epithelium through alternative modalities such as electrical or magnetic stimulation for improved accessibility. Future research should also explore the cognitive effects of intranasal air-puff stimulation in individuals with chronic sleep deprivation, such as those involved in shift work or military operations. Furthermore, it is recommended that future studies assess various stimulation parameters, such as the frequency and duration of intranasal air-puffing, and utilize fMRI to investigate the impact of intranasal air-puffing on brain activity.

## 5. Conclusions

The results of the present study indicated that the reduced cognitive capacity and cognitive performance in the brain due to sleep deprivation can be significantly improved by mechanical stimulation of the olfactory epithelium neurons. However, further studies are needed to determine the optimal stimulation frequency and assess the duration of its effects.

## Figures and Tables

**Table 1:** Demographic characteristics of the subjects.

Groups		*n*	Gender		Age (Years Old)	Weight (kg)
			Male (*n*)	Female (*n*)		
Control		8	3	5	27.50 ± 1.33	72 ± 9.34
Treatment	Subgroup 1	7	4	3	27.86 ± 1.908	75.57 ± 11.62
	Subgroup 2	6	3	3	26.67 ± 1.726	74.33 ± 8.441
	Subgroup 1 + Subgroup 2	13	7	6	27.31 ± 1.26	75.00 ± 7.08

## Data Availability

The data that support the findings of this study are available from the corresponding author upon reasonable request. The data are not publicly available due to privacy restrictions.

## Supplementary Materials

The following supporting information can be downloaded at: https://www.mdpi.com/article/10.3390/brainsci14040378/s1.
