# Supplementary material for: Olfactory Epithelium Stimulation Using Rhythmic Nasal Air-Puffs Improves the Cognitive Performance of Individuals with Acute Sleep Deprivation
Source: Brain Sci. 2024 Apr 13;14(4):378. doi: 10.3390/brainsci14040378 (PMC11048381; doi:10.3390/brainsci14040378)
Supplement: Supplementary file 1 [file brainsci-14-00378-s001.zip › brainsci-2923050-supplementary.pdf]

Olfactory epithelium stimulation using rhythmic nasal air-puffs improves the cognitive performance of individuals with acute sleep deprivation-

Supplementary material

Hanieh Riazi, Milad Nazari, Mohammad Reza Raoufy, Javad Mirnajafi-Zadeh, Amir Shojaei

Table of contents:

Table S1. Persian version of cognitive disability questionnaire

Table S2. English translated version of cognitive disability questionnaire

Table S3. English translated version of the brief questionnaire to assess participants' adherence to experimental rules

Table S4. Quantitative data of NST in subjects from the control and treatment groups

Table S5. Quantitative data of various numerical distances in NST for subjects in the control and treatment groups

Table S6. Quantitative data of the NST for the treatment group according to different flow rates of intranasal air- puff application (5.5 and 6 L/min)

Table S7. Quantitative data of NST conducted following intranasal air-puff application in subgroups 1 and 2 of the treatment group

Table S8. Quantitative data of EEG delta power for different electrodes in states of treatment group subjects

Table S9. Quantitative data of EEG theta power for different electrodes in states of treatment group subjects

Table S10. Quantitative data of EEG alpha power for different electrodes in states of treatment group subjects

Table S11. Quantitative data of EEG beta power for different electrodes in states of treatment group subjects

Table S12. Quantitative data of EEG slow gamma power for different electrodes in states of treatment group subjects

Table S13. Quantitative data of EEG medium gamma power for different electrodes in states of treatment group subjects

Table S14. Quantitative data of EEG fast gamma power for different electrodes in states of treatment group subjects

Table S15. Quantitative analysis of Higuchi's fractal dimension of EEG across various electrodes in states of treatment group subjects

Table S16. Quantitative analysis of Katz's fractal dimension of EEG across various electrodes in states of treatment group subjects

Table S17. Quantitative analysis of sample entropy of EEG across various electrodes in states of treatment group subjects

Table S18. Results of ANOVA analysis of EEG delta power across various electrodes in treatment group subjects

Table S19. Results of ANOVA analysis of EEG theta power across various electrodes in treatment group subjects

Table S20. Results of ANOVA analysis of EEG alpha power across various electrodes in treatment group subjects

Table S21. Results of ANOVA analysis of EEG beta power across various electrodes in treatment group subjects

Table S22. Results of ANOVA analysis of EEG slow gamma power across various electrodes in treatment group subjects

Table S23. Results of ANOVA analysis of EEG medium gamma power across various electrodes in treatment group subjects

Table S24. Results of ANOVA analysis of EEG fast gamma power across various electrodes in treatment group subjects

Table S25. Results of ANOVA analysis of Higuchi's fractal dimension of EEG across various electrodes in treatment group subjects

Table S26. Results of ANOVA analysis of Katz's fractal dimension of EEG across various electrodes in treatment group subjects

Table S27. Results of ANOVA analysis of sample entropy of EEG across various electrodes in treatment group subjects

Table S28. Specific p-values for statistical comparisons of intra-DMN cross-correlation among different states and No SD across various frequency bands

Table S29. Specific p-values for statistical comparisons of intra-DMN coherence among different states and No SD across various frequency bands

Figure S1. Comparison of age, weight, sleep quality index and cognitive disability index of subjects among control and treatment groups

Figure S2. Comparison of NST parameters among control and treatment group subjects on day 1 (before sleep deprivation)

Figure S3. Comparing the effect of applying nasal air-puffs on NST parameters at different numerical distances between control and treatment the groups following one night of PSD

Figure S4. Comparing the effect of nasal air-puffs flow rates (5.5 and 6 L/min) on subjects' performance in the NST following one night of PSD

Figure S5. The effect of transposing the timing of nasal air-puffs administration on the performance of subjects in NST following one night of PSD

Figure S6. Correlation between the effectiveness of nasal air-puffing in improving the performance of subjects in NST following PSD and their pre-existing sleep quality and cognitive disability indices

Supplementary materials and methods

Table S1. Persian version of cognitive disability questionnaire

رديف	سوالات	تقريباً هرگز	به ندرت	گاهي اوقات	اغلب	تقريباً هميشه
1	به خاطر آوردن كارهايي كه قصد انجام دادن آنها را دارم، مشكل است.					
2	به خاطر آوردن وقايعي كه هفته گذشته برايم روي داده است، مشكل است.					
3	اسامي افرادي كه هر روز با آنها سر و كار دارم، يادم مي‌رود.					
4	شناسايي افرادي كه قبلاً ملاقات كرده‌ام ، مشكل است.					
5	من فراموش مي‌كنم به چه منظوري از خانه بيرون آمده‌ام.					
6	در گفتگو موضوع مكالمه را فراموش مي‌كنم و دائم حاشيه مي‌روم.					
7	انجام همزمان دو كار برايم مشكل است و حواسم را پرت مي‌كند.					
8	تغير عادت موجب احساس ناراحتي و تشويش در من مي‌شود.					
9	يادگيري مهارت‌هاي جديد برايم سخت است.					
10	من با كوچك‌ترين صدايي تمركزم از بين مي‌رود.					
11	من در جمع نظرات نامناسبي ارائه ميدهم كه بعداً متوجه مي‌شوم كه بهتر بود ناگفته باقي مي‌ماند.					
12	خيلي وقت ها به ياد كارهايي كه قبلاً انجام داده‌ام مي‌افتم، از نسنجيده بودن آنها تعجب مي‌كنم.					
13	پاداش سريع كم را بر پاداش زياد ديرتر ترجيح مي‌دهم.					
14	سرعت انجام كارها از دقت آنها برايم مهمتر است.					
15	در تصميمگيري حوصله سبك و سنگين كردن شرايط را ندارم و دم دست ترين گزينه را انتخاب مي‌كنم.					
16	انتظار كشيدن برايم سخت است. مثلا يك دقيقه پشت چراغ قرمز ايستادن برايم خيلي طولاني به نظر مي‌آيد.					
17	در حين انجام كار نمي توانم بين ابعاد مهم و غيرمهم كار تفاوت قائل شوم و هر كاري دم دستم بود انجام مي دهم.					
18	براي دستيابي به اهداف بلند مدت خود، نمي توانم اهداف كوتاه مدت و نقشه رسيدن به آنها را ترسيم كنم.					
19	من برنامه ريزي طولاني مدتي براي آينده خود ندارم.					
20	برنامه ريزي كارهاي روزانه برايم دشوار است.					
21	من نميتوانم مدت زيادي به حرف هاي افرادي كه شمرده و كند حرف ميزنند، گوش كنم.					
22	اگر بخواهم شير اجاق گاز را كم كنم، معمولاً آن را خاموش مي‌كنم.					
23	گوش دادن به يك سخنراني تلويزيوني به طور كامل، برايم خسته كننده است.					
24	درصورتي كه يك فرد در يك جلسه اجتماعي معذب باشد، من كاري مي‌كنم كه فرد حس راحت تري پيدا كند.					
25	به اينكه ديگران به حرفهايم گوش كنند، توجه مي‌كنم.					
26	مي توانم منظور افراد را با نگاه كردن به آنها متوجه شوم.					
27	من فراموش مي كنم وسايلم را كجا گذاشته‌ام و دائم دنبال وسايلم مي‌گردم.					
28	خيلي وقتها تصميمي مي‌گيرم كه عواقب آن را در نظر نگرفته و بعداً پشيمان مي‌شوم.					
29	بيش از ده دقيقه نمي‌توانم روي يك موضوع (مثلا مطالعه) تمركز كنم.					
30	نمي‌توانم در حين گوش دادن به يك سخنراني از آن يادداشت بردارم.					

Table S2. English translated version of cognitive disability questionnaire

Row	Questions	Almost never	Rarely	Sometimes	Mostly	Almost always
1	It is difficult to remember the things that I intend to do.					
2	It is difficult to remember the events which happened to me last week.					
3	I forget the names of the people whom I deal with every day.					
4	It is difficult to recognize people whom I have met before.					
5	I forget why I left the house.					
6	In the conversation, I forget the topic and always quibble.					
7	It is difficult for me to do two things at the same time and it distracts me.					
8	Changing the habit makes me feel uncomfortable and confused.					
9	Learning new skills is difficult for me.					
10	I lose my concentration at the quiet sound.					
11	I often give unsuitable comments in the group that I later realize I should not have said.					
12	Many times, I remember the things that I have done before, but I am often surprised by their thoughtlessness.					
13	I prefer a quick and small reward over a delayed but larger reward.					
14	The speed of completing tasks is more important than their accuracy.					
15	When making decisions, I don't have the patience to ponder the situation. Instead, I opt for the most convenient and available choice.					
16	It is hard for me to wait. For example, standing at a red light for a minute seems too long to me.					
17	While doing work, I can't differentiate between important and unimportant aspects of work and I do whatever I can.					
18	I can't set short-term goals and map out how to achieve them in order to reach my long-term goals.					
19	I have no long-term plans for my future.					
20	It is difficult for me to plan my daily activities.					
21	I can't listen for a long time to people who articulate and speak slowly.					
22	If I want to decrease the heat on the stove, I usually turn it off.					
23	Listening to an entire televised lecture is boring for me.					
24	If a person is uncomfortable in a social meeting, I do something to make the person feel more comfortable.					
25	I pay attention to others listening to my speaking.					
26	I can understand what people mean by looking at them.					
27	I forget where I put my things and I am looking for them.					
28	I often make a decision without considering its outcome and I regret later.					
29	I can't concentrate on a subject (for example, study) for more than ten minutes.					
30	I can't take notes while listening to a lecture.					

Table S3. English translated version of the brief questionnaire to assess participants' adherence to experimental rules

This questionnaire is about your behavior in the last 24 h. Please answer the following questions carefully.

Row	Questions	Answers
1	What time did you go to bed at?	
2	How many minutes did it take you from the time you lay in bed to the time you fell asleep?	
3	Did you sleep continuously during sleep or did you wake up to do something (If you wake up, how many times and for how long)?	
4	Did you take a nap or sleep in the last 24 h (if yes, how many times, at what time and for how long)?	
5	What time did you wake up?	
6	Have you consumed caffeinated beverages (tea or coffee) in the last 24 h? (If the answer is positive, how many times and to what extent)?	
7	Have you consumed alcoholic beverages in the last 24 h? (If the answer is positive, how many times and to what extent)?	
8	Did you do vigorous sports in the last 24 h or not? (If the answer is positive, how many times and to what extent)?	

Supplementary results

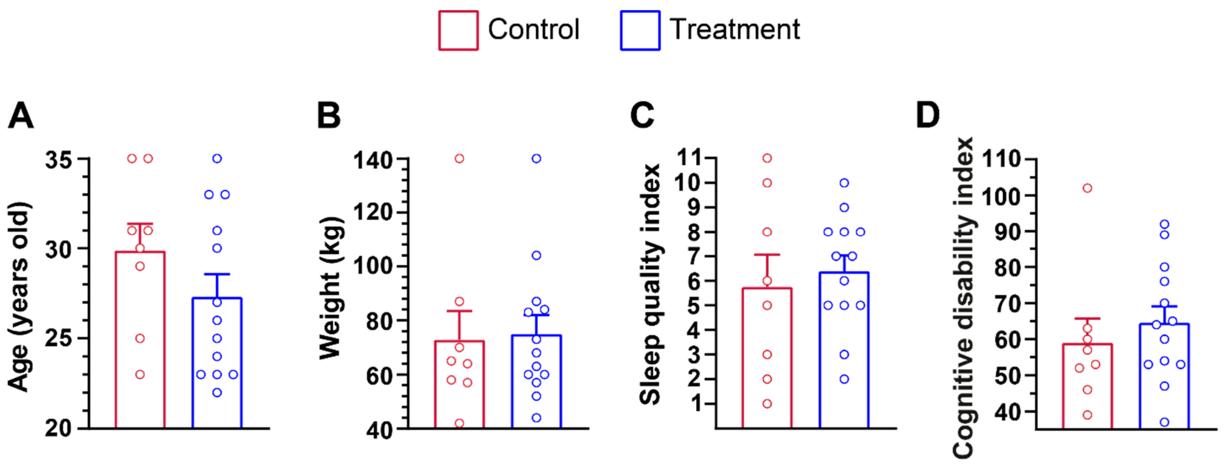

Figure S1. Comparison of age (A), weight (B), sleep quality index (C) and cognitive disability index (D) of subjects among control and treatment groups. Unpaired t-test in A and C and Mann-Whitney U test in B and D; mean±SEM.

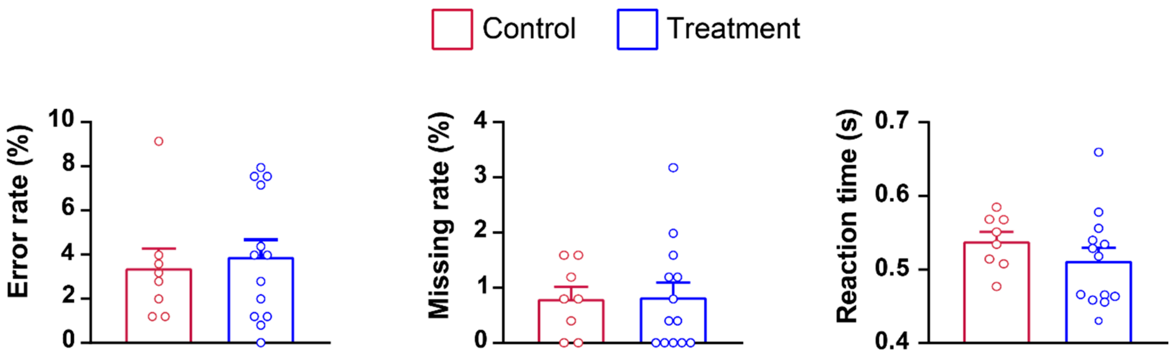

Figure S2. Comparison of NST parameters among control and treatment group subjects on day 1 (before sleep deprivation). Unpaired t-test; mean±SEM.

Table S4. Quantitative data of NST in subjects from the control and treatment groups. Mean±SEM.

Experimental Group	State	Error rate	Missing rate	Reaction time
Control **(n=8)**	No SD	3.37301±0.8999	0.79365±0.22498	0.53801±0.01295
	SD+Routine respiration	5.655±1.229	0.397±0.07499	0.501±0.01563
	SD+Oral respiration	6.349±1.152	1.687±0.6527	0.501±0.01816
Treatment **(n=13)**	No SD	3.87668±0.79257	0.82418±0.27131	0.51168±0.01772
	SD+Air-puff	3.63248±0.93264	2.04518±1.23120	0.49954±0.01738
	SD+nasal respiration	4.63980±0.90466	1.34310±0.51922	0.49923±0.01534

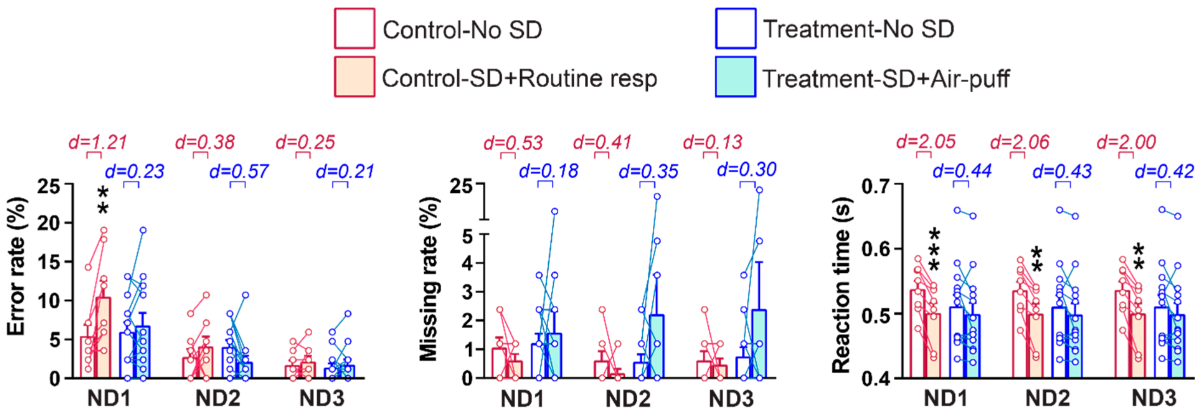

Figure S3. Comparing the effect of applying nasal air-puffs on NST parameters at different numerical distances between control and treatment the groups following one night of PSD. ** p<0.01 and *** p<0.001; two-way repeated measures ANOVA; d shows Cohen's d number; mean±SEM.

Table S5. Quantitative data of various numerical distances in NST for subjects in the control and treatment groups. Mean±SEM.

Experimental Group	State	Error rate			Missing rate			Reaction time		
		ND1	ND2	ND3	ND1	ND2	ND3	ND1	ND2	ND3
Control (n=8)	No SD	5.50595± 1.4569	2.82738± 1.0294	1.78571± 0.5952	1.04167± 0.3512	0.59524± 0.3181	0.59524± 0.3181	0.53801± 0.0129	0.53637± 0.0131	0.53681± 0.0130
	SD+ Routine respiration	10.56548±2.0550	4.16667± 1.2727	2.23214± 0.7268	0.59524± 0.2250	0.14881± 0.1488	0.44643± 0.2178	0.50112± 0.0156	0.50045± 0.0154	0.50059± 0.0158
Treatment (n=13)	No SD	6.04396± 1.2314	4.12088± 0.8902	1.46520± 0.5417	1.19048± 0.3302	0.54945± 0.2563	0.73260± 0.3172	0.51168± 0.0177	0.51116± 0.0177	0.51136± 0.0178
	SD+ Air-puff	6.86813± 1.6240	2.19780± 0.7842	1.83150± 0.6688	1.55678± 0.7430	2.19780± 1.3736	2.38095± 1.6287	0.49954± 0.0174	0.49894± 0.0174	0.49939± 0.0173

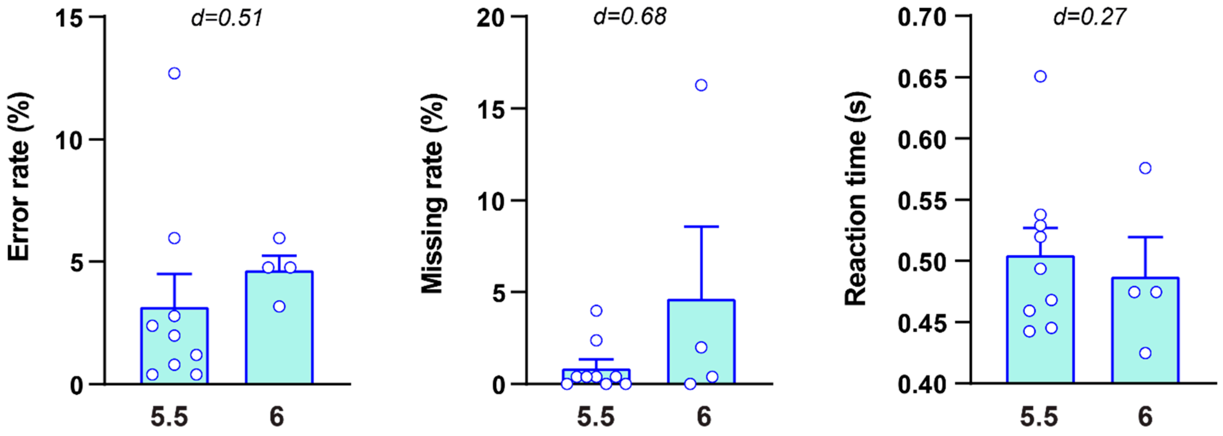

Figure S4. Comparing the effect of nasal air-puffs flow rates (5.5 and 6 L/min) on subjects' performance in the NST following one night of PSD. Unpaired t-test; mean±SEM.

Table S6. Quantitative data of the NST for the treatment group according to different flow rates of intranasal air- puff application (5.5 and 6 L/min). Mean±SEM.

**Parameter**	**5 (L/min)**	**6 (L/min)**
Error rate	3.175±1.321	4.663±0.5699
Missing rate	0.889±0.4572	4.663±3.893
Reaction time	0.505±0.02178	0.487±0.03178

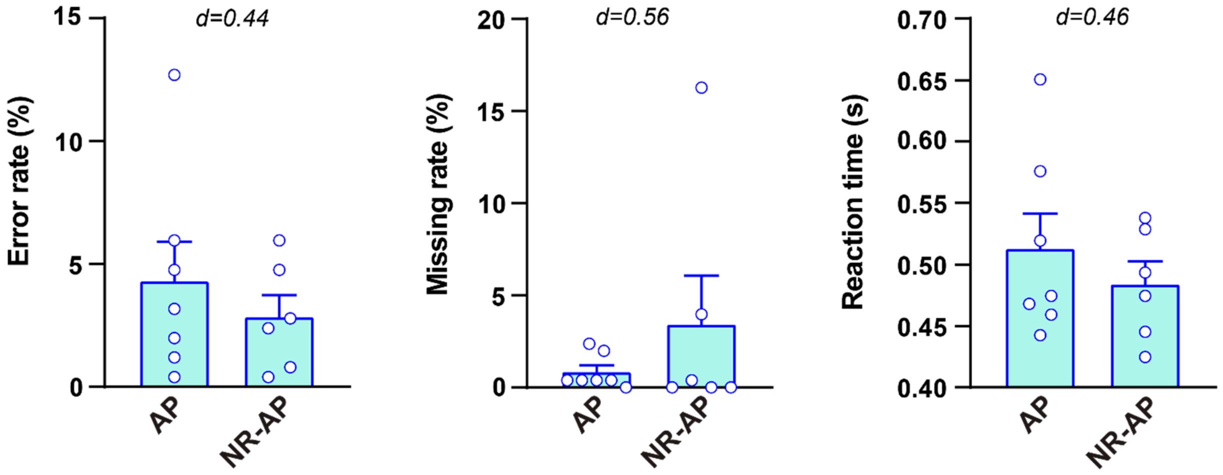

Figure S5. The effect of transposing the timing of nasal air-puffs administration on the performance of subjects in NST following one night of PSD. “AP” denotes data from subgroup 1, in which nasal air-puffs were applied around 23 min before nasal respiration, and “NR-AP” presents data from subgroup 2, where air-puffs applied around 20 min after the cessation of nasal respiration. paired t-test; mean±SEM.

Table S7. Quantitative data of NST conducted following intranasal air-puff application in subgroups 1 and 2 of the treatment group. Mean±SEM.

Experimental Group	State	Error rate	Missing rate	Reaction time
Treatment **(n=13)**	AP	4.308±1.581	0.850±0.3510	0.513±0.02859
	NR-AP	2.844±0.8898	3.439±2.644	0.484±0.01837

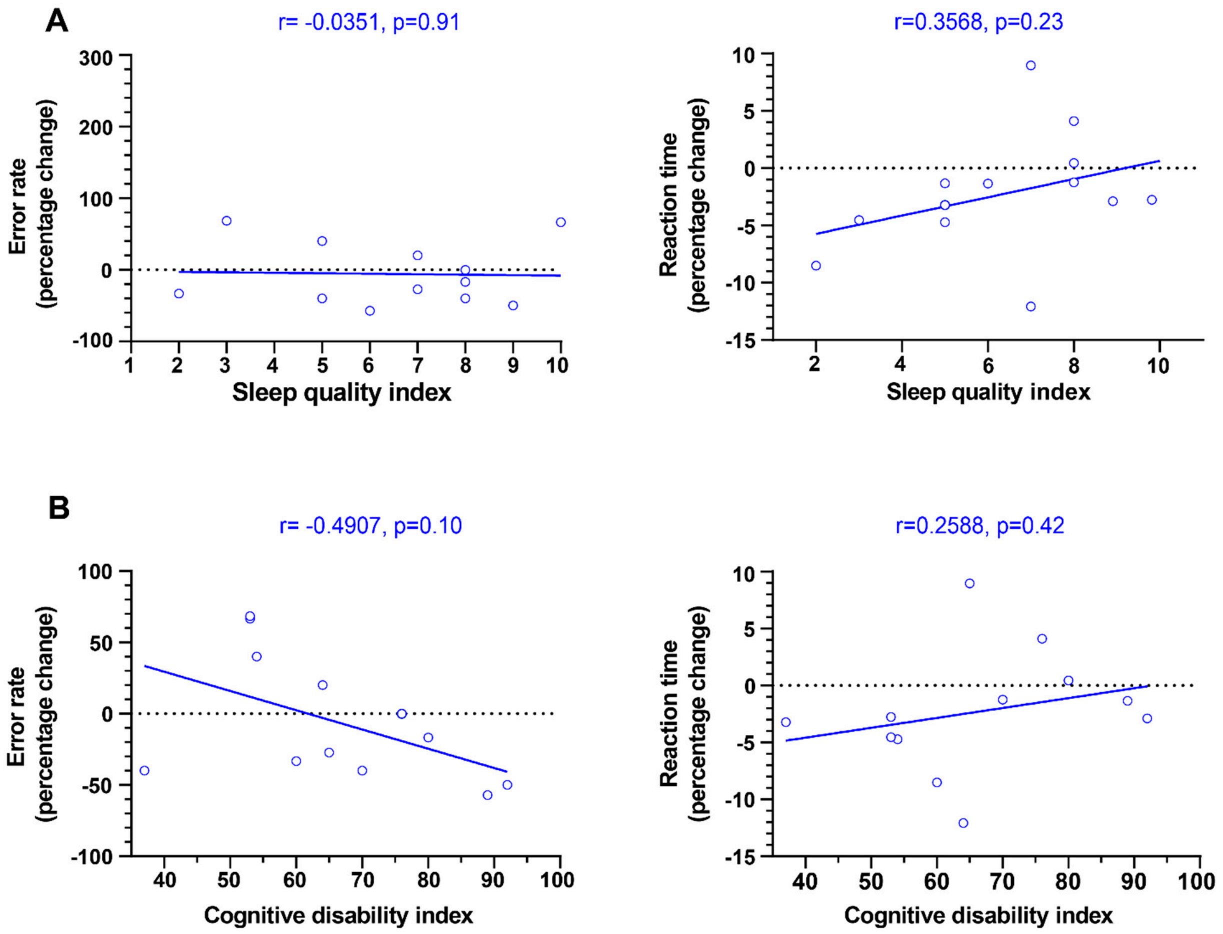

Figure S6. Correlation between the effectiveness of nasal air-puffing in improving the performance of subjects in NST following PSD and their pre-existing sleep quality (A) and cognitive disability (B) indices. Pearson correlation; mean±SEM.

Table S8. Quantitative data of EEG delta power for different electrodes in states of treatment group subjects. Mean±SEM.

Electrodes	No SD	SD	SD+Air-puff	SD+Nasal resp
Fp1	2.27366±0.37348	3.26204±0.63104	1.65448±0.28945	2.55536±0.52649
Fpz	1.27453±0.23389	1.72391±0.52128	1.09597±0.1386	2.28767±0.87106
AFz	2.42117±0.46188	2.54954±0.2678	1.64885±0.19032	2.69822±0.28357
Fp2	1.05341±0.134	1.20233±0.16675	1.02033±0.13285	1.338±0.25595
F7	0.99179±0.15321	1.39273±0.24954	0.98928±0.12705	1.17308±0.15241
F3	2.00565±0.36169	2.37648±0.30664	1.58411±0.18427	1.94708±0.27903
Fz	1.39835±0.25122	1.45354±0.21271	1.21563±0.18535	1.22739±0.15517
F4	2.02294±0.32804	3.34573±0.76611	1.97146±0.37103	3.11721±1.15686
F8	2.2486±0.33008	2.48472±0.30041	1.69849±0.25264	2.61695±0.46471
FC5	1.45529±0.55798	1.07835±0.10503	0.88503±0.08618	1.01202±0.1284
FC1	1.95698±0.41424	1.87642±0.25905	1.53357±0.25677	1.87758±0.34203
FC2	1.14254±0.19694	1.18496±0.17317	1.00707±0.16557	1.09767±0.16372
FC6	0.9115±0.16164	1.13274±0.22449	0.96813±0.17185	0.99462±0.16647
C3	1.0167±0.21968	1.30271±0.38842	0.91776±0.18992	1.194±0.27775
Cz	0.99162±0.13804	1.06586±0.14806	0.85404±0.13868	0.98669±0.13603
C4	1.22436±0.24505	1.38345±0.2926	1.09977±0.16929	1.34522±0.29097
CP5	1.24166±0.19844	1.28608±0.16263	1.0832±0.15401	1.22756±0.15491
CP1	1.20462±0.1949	1.26079±0.24018	0.95857±0.13333	1.19514±0.18044
CPz	0.98018±0.2371	0.98607±0.26388	0.5964±0.09025	0.80626±0.12695
CP2	0.9814±0.13722	1.01334±0.16431	0.8006±0.12161	1.00163±0.12541
CP6	1.01543±0.20922	1.32529±0.35972	0.97553±0.16818	1.23374±0.28742
P7	1.38199±0.32303	1.09785±0.1967	0.94096±0.19312	1.17016±0.20786
P3	1.86914±0.25618	2.12537±0.40281	1.81974±0.21334	1.93582±0.22317
Pz	1.47263±0.20835	1.36022±0.16321	1.08704±0.14731	1.38522±0.17937
P4	1.64816±0.36758	1.43786±0.23168	1.18151±0.18727	1.66324±0.20936
P8	2.04701±0.22499	2.25137±0.4764	1.91737±0.41174	2.37559±0.37903
O1	2.20196±0.27073	2.12383±0.25595	1.77622±0.2607	2.09723±0.27541
POz	1.64092±0.25699	1.71422±0.27446	1.42983±0.24457	1.59857±0.26205
O2	2.25835±0.29276	2.14404±0.24244	1.73102±0.22333	2.07052±0.23556
T7	1.58244±0.24555	1.69348±0.27692	1.22375±0.18146	1.57577±0.17659
T8	2.09371±0.55265	1.76548±0.36853	1.35805±0.27748	1.58168±0.35264

Table S9. Quantitative data of EEG theta power for different electrodes in states of treatment group subjects. Mean±SEM.

Electrodes	No SD	SD	SD+Air-puff	SD+Nasal resp
**Fp1**	0.6173±0.1045	0.73657±0.10209	0.55997±0.0878	0.70046±0.13159
**Fpz**	0.50147±0.06509	0.5592±0.06796	0.49652±0.06726	0.58843±0.06906
**AFz**	0.82266±0.14243	0.88707±0.13025	0.65121±0.08329	0.92677±0.11469
**Fp2**	0.75531±0.16995	0.81592±0.17551	0.71904±0.16274	0.81536±0.15028
**F7**	0.40464±0.04581	0.46315±0.07141	0.37641±0.05568	0.45604±0.06867
**F3**	0.61049±0.09531	0.79651±0.10477	0.62022±0.08994	0.76718±0.14741
**Fz**	0.70718±0.11345	0.79003±0.10293	0.65093±0.09589	0.84933±0.12822
**F4**	0.74185±0.08696	0.98729±0.13618	0.84001±0.12536	1.02839±0.14617
**F8**	0.63064±0.10286	0.77735±0.09824	0.58676±0.08524	0.80602±0.13606
**FC5**	0.44697±0.0511	0.49104±0.05218	0.44339±0.04928	0.5305±0.06365
**FC1**	0.64916±0.08139	0.70111±0.07037	0.65648±0.07326	0.74337±0.09102
**FC2**	0.65641±0.17648	0.68318±0.14983	0.66288±0.15979	0.6621±0.14924
**FC6**	0.45504±0.0866	0.52311±0.08747	0.47016±0.08259	0.52899±0.10111
**C3**	0.39268±0.10806	0.40583±0.13104	0.37119±0.10719	0.39397±0.11432
**Cz**	0.42171±0.09321	0.4697±0.09234	0.40391±0.08198	0.4427±0.08633
**C4**	0.5753±0.09498	0.66676±0.11306	0.6181±0.1197	0.71215±0.12046
**CP5**	0.61187±0.14229	0.68756±0.12591	0.60894±0.11245	0.63832±0.11431
**CP1**	0.52356±0.08998	0.55998±0.07964	0.49063±0.07653	0.53305±0.07615
**CPz**	0.29546±0.036	0.35876±0.05583	0.30906±0.04866	0.33848±0.05473
**CP2**	0.45743±0.11953	0.50704±0.09608	0.45052±0.10071	0.49928±0.09066
**CP6**	0.49248±0.09574	0.61311±0.11291	0.53106±0.10848	0.62627±0.12724
**P7**	0.55813±0.0824	0.63095±0.08536	0.56844±0.0999	0.66651±0.1013
**P3**	0.7662±0.08364	0.87356±0.14256	0.79227±0.10371	0.92468±0.14293
**Pz**	0.54602±0.0867	0.58854±0.07452	0.51089±0.07488	0.57985±0.07841
**P4**	0.57564±0.09844	0.69819±0.12557	0.57533±0.10264	0.72475±0.14206
**P8**	0.70821±0.08583	0.87793±0.09229	0.80131±0.10132	0.91664±0.13573
**O1**	0.79879±0.10657	0.90383±0.11378	0.7741±0.10615	0.87963±0.1095
**POz**	0.68017±0.09085	0.76648±0.10336	0.65293±0.10441	0.82209±0.11795
**O2**	0.90023±0.12205	1.02733±0.12862	0.8947±0.13277	1.00225±0.13243
**T7**	0.64062±0.06218	0.74554±0.13269	0.57747±0.09017	0.75739±0.11833
**T8**	0.63112±0.13014	0.73265±0.1162	0.63016±0.11062	0.65253±0.11531

Table S10. Quantitative data of EEG alpha power for different electrodes in states of treatment group subjects. Mean±SEM.

Electrodes	No SD	SD	SD+Air-puff	SD+Nasal resp
Fp1	0.42562±0.09959	0.82912±0.30454	0.71177±0.27877	0.67365±0.19616
Fpz	0.36629±0.08746	0.52437±0.19089	0.37195±0.08012	0.36467±0.0663
AFz	0.88313±0.36461	0.97964±0.37214	0.90893±0.42004	1.01426±0.43048
Fp2	0.404±0.11343	0.56787±0.1954	0.41307±0.10147	0.48777±0.12024
F7	0.64013±0.2148	0.76379±0.26305	0.79233±0.2991	0.83746±0.31578
F3	0.42989±0.11122	0.86524±0.31245	0.55724±0.15314	0.86913±0.3621
Fz	0.4066±0.0987	0.67368±0.28149	0.41375±0.09134	0.59724±0.20176
F4	0.45856±0.12894	0.68681±0.25846	0.45903±0.11183	0.55466±0.13767
F8	0.41212±0.08769	0.7602±0.33644	0.43698±0.08651	0.57429±0.14321
FC5	0.34878±0.08433	0.46843±0.1322	0.37422±0.08539	0.44397±0.09487
FC1	0.44831±0.10488	0.52063±0.11628	0.46278±0.09402	0.4693±0.11227
FC2	0.47871±0.15621	0.60542±0.18953	0.49563±0.13433	0.57374±0.16457
FC6	0.56301±0.24716	0.58448±0.2479	0.60982±0.28927	0.68948±0.31689
C3	0.59524±0.20456	0.72863±0.27777	0.75593±0.30392	0.78304±0.28785
Cz	0.3634±0.14695	0.51915±0.19833	0.39057±0.12834	0.44338±0.15669
C4	1.00661±0.3712	1.26025±0.51502	1.28986±0.59443	1.48138±0.66726
CP5	0.58204±0.22661	0.62462±0.22019	0.59017±0.23694	0.62357±0.22075
CP1	0.72901±0.35835	0.92987±0.39151	0.81956±0.42537	0.90569±0.44748
CPz	0.5822±0.28579	0.66432±0.32024	0.62488±0.2699	0.67796±0.35077
CP2	0.70591±0.32011	0.89562±0.36061	0.86687±0.42207	1.03574±0.52314
CP6	0.60041±0.18244	0.72237±0.21744	0.65842±0.19028	0.74521±0.23973
P7	0.84725±0.27919	1.47442±0.59933	1.35857±0.72574	1.75562±1.01445
P3	1.62756±0.47319	1.7012±0.5017	1.56241±0.50741	1.85471±0.68754
Pz	0.37338±0.05192	0.74763±0.39774	0.48236±0.10666	0.51581±0.18259
P4	1.56144±0.64755	1.88966±1.04329	3.30764±1.45695	3.37707±1.81117
P8	1.05029±0.34714	1.19329±0.35898	1.06369±0.33879	1.42159±0.57122
O1	1.11198±0.18211	1.46165±0.38148	1.22368±0.37386	1.39319±0.55875
POz	0.78139±0.16578	1.09888±0.37366	0.76842±0.12775	0.84645±0.19583
O2	1.20422±0.23357	1.25519±0.24487	1.0306±0.15174	0.98001±0.14191
T7	1.09318±0.39192	1.10296±0.43315	1.0247±0.39862	1.14202±0.44854
T8	0.80187±0.31946	1.1948±0.50683	1.5545±0.67549	1.43339±0.57238

Table S11. Quantitative data of EEG beta power for different electrodes in states of treatment group subjects. Mean±SEM.

Electrodes	No SD	SD	SD+Air-puff	SD+Nasal resp
**Fp1**	0.14806±0.02059	0.13992±0.01765	0.18657±0.02391	0.15577±0.02284
**Fpz**	0.1932±0.05589	0.17415±0.03577	0.24467±0.04927	0.19231±0.03056
**AFz**	0.18025±0.02338	0.14798±0.01226	0.22781±0.03032	0.19569±0.01823
**Fp2**	0.14669±0.03755	0.15518±0.04168	0.16726±0.04259	0.15618±0.03842
**F7**	0.17399±0.03702	0.13137±0.02233	0.18314±0.03435	0.18125±0.05467
**F3**	0.23132±0.048	0.20613±0.04297	0.23655±0.04449	0.18751±0.02687
**Fz**	0.11888±0.01504	0.10784±0.01683	0.12121±0.01813	0.12476±0.02406
**F4**	0.12958±0.01876	0.14579±0.02684	0.1528±0.02658	0.14484±0.02575
**F8**	0.21299±0.0481	0.15753±0.0255	0.24393±0.0652	0.15475±0.02747
**FC5**	0.1339±0.01677	0.14757±0.01941	0.21855±0.03769	0.15909±0.0219
**FC1**	0.20585±0.03582	0.16171±0.01792	0.24325±0.03738	0.19189±0.03457
**FC2**	0.15373±0.04047	0.15118±0.0361	0.18391±0.03982	0.14952±0.03509
**FC6**	0.15453±0.02953	0.10514±0.01299	0.15858±0.02207	0.13504±0.03067
**C3**	0.1362±0.0204	0.11825±0.01947	0.17271±0.02691	0.14885±0.03201
**Cz**	0.08431±0.01242	0.07902±0.0137	0.09603±0.01908	0.10006±0.02209
**C4**	0.20047±0.04417	0.16605±0.03308	0.21882±0.03369	0.18208±0.03241
**CP5**	0.13297±0.0406	0.13162±0.03917	0.14564±0.03876	0.14312±0.04088
**CP1**	0.09751±0.01688	0.09629±0.01957	0.11781±0.02565	0.10756±0.02035
**CPz**	0.20118±0.04987	0.1434±0.02606	0.18453±0.03795	0.15926±0.03254
**CP2**	0.15725±0.05937	0.14689±0.04748	0.17528±0.05158	0.15581±0.0469
**CP6**	0.12806±0.01825	0.11391±0.0152	0.14194±0.02089	0.12044±0.01657
**P7**	0.19578±0.0513	0.16745±0.04344	0.1953±0.04253	0.17781±0.041
**P3**	0.28493±0.04761	0.2383±0.04327	0.31361±0.05653	0.26129±0.04316
**Pz**	0.11821±0.01778	0.11267±0.023	0.15039±0.03535	0.12905±0.0278
**P4**	0.13981±0.02003	0.14415±0.02557	0.20923±0.04073	0.17494±0.03339
**P8**	0.23086±0.03509	0.19594±0.03723	0.25113±0.04323	0.21199±0.02981
**O1**	0.30024±0.03673	0.28216±0.03716	0.33317±0.04121	0.29574±0.04203
**POz**	0.17087±0.0279	0.14848±0.02501	0.16649±0.0274	0.16823±0.02651
**O2**	0.35019±0.07514	0.33924±0.06239	0.38021±0.06524	0.32357±0.05276
**T7**	0.32126±0.06658	0.19222±0.03085	0.25241±0.03247	0.24146±0.03573
**T8**	0.23923±0.0619	0.19287±0.03747	0.22616±0.03379	0.17781±0.0275

Table S12. Quantitative data of EEG slow gamma power for different electrodes in states of treatment group subjects. Mean±SEM.

Electrodes	No SD	SD	SD+Air-puff	SD+Nasal resp
Fp1	0.0611±0.03297	0.0443±0.02017	0.05226±0.01698	0.03809±0.01165
Fpz	0.04433±0.01181	0.02921±0.00704	0.0478±0.01148	0.03801±0.00907
AFz	0.04616±0.00989	0.03189±0.00368	0.05834±0.01353	0.0413±0.00737
Fp2	0.01606±0.00167	0.0138±0.00125	0.01516±0.00127	0.01378±0.0011
F7	0.04462±0.00781	0.02414±0.00437	0.04352±0.01002	0.04448±0.01968
F3	0.05887±0.01972	0.0434±0.01691	0.05761±0.01856	0.03651±0.01219
Fz	0.02381±0.00549	0.01438±0.00115	0.01821±0.00317	0.01669±0.00293
F4	0.02174±0.0039	0.01653±0.00191	0.01732±0.00176	0.01567±0.00147
F8	0.06038±0.02075	0.03796±0.01223	0.06396±0.02364	0.03615±0.00909
FC5	0.02805±0.00447	0.02375±0.00369	0.04879±0.01422	0.02541±0.00359
FC1	0.05155±0.01381	0.03365±0.00533	0.05785±0.01315	0.04246±0.01218
FC2	0.01973±0.00264	0.01753±0.00239	0.02566±0.00522	0.01641±0.00155
FC6	0.04678±0.01335	0.01882±0.00236	0.03962±0.00885	0.02673±0.00835
C3	0.03595±0.00875	0.01957±0.00349	0.04415±0.01425	0.0282±0.01087
Cz	0.01464±0.00266	0.01218±0.00199	0.01524±0.00223	0.01175±0.00116
C4	0.04777±0.02112	0.02446±0.00362	0.04589±0.00944	0.02587±0.00407
CP5	0.01569±0.00272	0.01376±0.00249	0.0157±0.00227	0.01419±0.00228
CP1	0.01388±0.00177	0.01143±0.00122	0.01323±0.0014	0.0112±0.00074
CPz	0.06545±0.02283	0.02716±0.00593	0.04114±0.01063	0.03082±0.00885
CP2	0.01482±0.00307	0.01275±0.00191	0.01602±0.00267	0.01163±0.00089
CP6	0.05116±0.02008	0.03989±0.01599	0.05351±0.01886	0.02627±0.0068
P7	0.03884±0.02036	0.02172±0.00578	0.0321±0.00942	0.01853±0.003
P3	0.06916±0.01501	0.03906±0.00814	0.0688±0.01552	0.03733±0.00472
Pz	0.0165±0.00202	0.01311±0.00147	0.01699±0.00233	0.01383±0.00162
P4	0.02157±0.00327	0.01886±0.0037	0.03888±0.01594	0.01745±0.00187
P8	0.03446±0.00618	0.0263±0.0038	0.03925±0.00697	0.02908±0.00549
O1	0.04505±0.00741	0.05013±0.01127	0.07018±0.01623	0.04231±0.00931
POz	0.02144±0.00294	0.01699±0.00152	0.02149±0.00233	0.02171±0.00492
O2	0.05057±0.0103	0.06196±0.01513	0.07141±0.01606	0.0473±0.01346
T7	0.08374±0.01908	0.03686±0.00735	0.06362±0.01228	0.0503±0.00937
T8	0.06504±0.02365	0.03745±0.00779	0.04954±0.00956	0.03123±0.00556

Table S13. Quantitative data of EEG medium gamma power for different electrodes in states of treatment group subjects. Mean±SEM.

Electrodes	No SD	SD	SD+Air-puff	SD+Nasal resp
**Fp1**	0.01805±0.01063	0.0124±0.00661	0.01537±0.00574	0.01062±0.0041
**Fpz**	0.0139±0.00352	0.01007±0.0027	0.01582±0.00422	0.01102±0.00208
**AFz**	0.01853±0.00748	0.00914±0.0012	0.01776±0.00376	0.01228±0.00195
**Fp2**	0.00455±0.00069	0.00297±0.00033	0.00384±0.00054	0.00304±0.00024
**F7**	0.01551±0.0025	0.01015±0.00343	0.01547±0.00368	0.01896±0.00981
**F3**	0.01442±0.00548	0.00689±0.00128	0.01203±0.00442	0.0095±0.0032
**Fz**	0.0088±0.00268	0.00368±0.00039	0.00534±0.0013	0.00451±0.00098
**F4**	0.00758±0.00257	0.00368±0.00053	0.00421±0.00059	0.0036±0.00035
**F8**	0.0194±0.00686	0.01075±0.00381	0.02341±0.01148	0.00872±0.00185
**FC5**	0.00906±0.00229	0.00804±0.00206	0.01626±0.00402	0.0064±0.00107
**FC1**	0.01955±0.00627	0.01094±0.00199	0.01821±0.00381	0.0133±0.0037
**FC2**	0.0057±0.00103	0.00442±0.00091	0.00713±0.00165	0.00394±0.00047
**FC6**	0.01664±0.0059	0.00756±0.00226	0.01683±0.00396	0.00954±0.00313
**C3**	0.01317±0.00429	0.00572±0.00143	0.0161±0.00637	0.00922±0.00448
**Cz**	0.00426±0.00126	0.00304±0.0008	0.00415±0.00093	0.00287±0.00039
**C4**	0.01921±0.01009	0.00754±0.00151	0.01626±0.00386	0.00798±0.00145
**CP5**	0.00347±0.00037	0.00259±0.00022	0.00337±0.00042	0.00268±0.00019
**CP1**	0.00366±0.00055	0.00258±0.00029	0.00333±0.00045	0.00241±0.0001
**CPz**	0.02421±0.00969	0.00851±0.00251	0.01578±0.00532	0.01054±0.00443
**CP2**	0.00511±0.00157	0.00379±0.00089	0.0051±0.0014	0.00291±0.00028
**CP6**	0.01917±0.00874	0.00985±0.00334	0.01534±0.00577	0.00776±0.00231
**P7**	0.00505±0.00057	0.00416±0.00049	0.00653±0.00099	0.00393±0.00032
**P3**	0.0253±0.00534	0.01249±0.00296	0.02243±0.00494	0.0116±0.0011
**Pz**	0.0042±0.00062	0.00284±0.00031	0.00393±0.00061	0.00282±0.00039
**P4**	0.0046±0.00063	0.00386±0.00071	0.00577±0.00097	0.00364±0.00042
**P8**	0.01404±0.00329	0.01086±0.00319	0.01487±0.00364	0.00969±0.00226
**O1**	0.02103±0.00542	0.01731±0.00341	0.02168±0.00625	0.01255±0.00242
**POz**	0.00431±0.00048	0.00352±0.00044	0.00476±0.00069	0.00339±0.00035
**O2**	0.01418±0.00288	0.01886±0.00498	0.02244±0.00548	0.01321±0.00397
**T7**	0.03198±0.00957	0.01653±0.00595	0.02444±0.0054	0.02152±0.00524
**T8**	0.01547±0.00681	0.01221±0.00261	0.01618±0.00324	0.00928±0.0019

Table S14. Quantitative data of EEG fast gamma power for different electrodes in states of treatment group subjects. Mean±SEM.

Electrodes	No SD	SD	SD+Air-puff	SD+Nasal resp
**Fp1**	0.00829±0.00467	0.00551±0.00269	0.00709±0.00266	0.00484±0.00167
**Fpz**	0.00732±0.00262	0.0037±0.00092	0.00656±0.002	0.00433±0.00087
**AFz**	0.01234±0.00717	0.00425±0.00051	0.00826±0.0016	0.00572±0.00087
**Fp2**	0.00222±0.00034	0.00143±0.00017	0.00182±0.00028	0.00157±0.00012
**F7**	0.00814±0.00142	0.005±0.00155	0.00766±0.00182	0.00912±0.00477
**F3**	0.00722±0.00263	0.00332±0.00055	0.00577±0.00226	0.00409±0.00128
**Fz**	0.00491±0.00196	0.00179±0.00018	0.00272±0.00063	0.00214±0.00046
**F4**	0.00494±0.00237	0.002±0.0004	0.00219±0.00037	0.00197±0.00024
**F8**	0.00914±0.00309	0.00516±0.00179	0.01209±0.0062	0.00424±0.00078
**FC5**	0.00538±0.00154	0.00378±0.0009	0.00805±0.00219	0.00327±0.00046
**FC1**	0.0119±0.00525	0.00515±0.00089	0.00879±0.00187	0.00616±0.00164
**FC2**	0.00327±0.00067	0.00229±0.00044	0.00362±0.00074	0.00216±0.00028
**FC6**	0.00949±0.0036	0.00384±0.00109	0.00856±0.0019	0.00478±0.00147
**C3**	0.00795±0.00301	0.00314±0.00075	0.0087±0.00351	0.00488±0.0023
**Cz**	0.00233±0.00081	0.0015±0.00041	0.00209±0.00048	0.00144±0.00019
**C4**	0.01212±0.00606	0.00566±0.00186	0.00964±0.00207	0.00604±0.00147
**CP5**	0.00216±0.00038	0.00144±0.00016	0.00201±0.00036	0.00154±0.00019
**CP1**	0.00212±0.00041	0.00139±0.0002	0.0018±0.00026	0.0013±0.00011
**CPz**	0.01251±0.00498	0.0047±0.00136	0.0085±0.00291	0.0056±0.00241
**CP2**	0.00291±0.00101	0.00191±0.00044	0.00277±0.00078	0.00154±0.00016
**CP6**	0.00652±0.00334	0.0033±0.00073	0.00659±0.003	0.0031±0.0005
**P7**	0.00274±0.00033	0.00224±0.00029	0.00343±0.00048	0.00218±0.00015
**P3**	0.01496±0.00324	0.00774±0.00215	0.01233±0.00276	0.00701±0.0006
**Pz**	0.00223±0.00038	0.00135±0.00014	0.00189±0.00033	0.00139±0.0002
**P4**	0.00496±0.00201	0.00457±0.00205	0.00817±0.00507	0.00273±0.00083
**P8**	0.007±0.00169	0.00558±0.00141	0.00758±0.00156	0.00541±0.00102
**O1**	0.00805±0.00149	0.00807±0.00155	0.0117±0.00267	0.00732±0.00142
**POz**	0.00305±0.00086	0.00197±0.0002	0.00276±0.00041	0.00259±0.00083
**O2**	0.0089±0.00194	0.00971±0.00259	0.01154±0.00271	0.00669±0.00176
**T7**	0.01612±0.00509	0.00705±0.0019	0.01212±0.00297	0.01058±0.00241
**T8**	0.00751±0.0035	0.00533±0.00108	0.00799±0.00187	0.00465±0.00089

Table S15. Quantitative analysis of Higuchi's fractal dimension of EEG across various electrodes in states of treatment group subjects. Mean±SEM.

Electrodes	No SD	SD	SD+Air-puff	SD+Nasal resp
Fp1	1.47142±0.03305	1.4301±0.03014	1.47838±0.0327	1.45831±0.03245
Fpz	1.5011±0.03069	1.45663±0.03139	1.49445±0.03227	1.43652±0.0274
Afz	1.50336±0.03785	1.43448±0.03643	1.49026±0.04406	1.45614±0.02642
Fp2	1.51153±0.03463	1.4362±0.03632	1.4856±0.0425	1.44917±0.03199
F7	1.50729±0.03508	1.45977±0.02264	1.50351±0.03268	1.48168±0.02964
F3	1.49485±0.03868	1.4404±0.03204	1.47248±0.03772	1.46429±0.02887
Fz	1.46271±0.03539	1.38685±0.02261	1.42937±0.03085	1.41268±0.02972
F4	1.51418±0.02877	1.46509±0.02821	1.51603±0.03744	1.4694±0.02621
F8	1.51913±0.03008	1.4615±0.02433	1.5057±0.03049	1.48003±0.02936
FC5	1.5239±0.03181	1.44344±0.02621	1.49779±0.03939	1.47014±0.02875
FC1	1.45148±0.0406	1.39236±0.02591	1.41866±0.03382	1.44291±0.03976
FC2	1.47894±0.02201	1.43276±0.02105	1.4889±0.03287	1.43645±0.0223
FC6	1.52301±0.02953	1.45694±0.02735	1.52087±0.03947	1.45193±0.02969
C3	1.56296±0.03454	1.48441±0.03352	1.53032±0.04333	1.49079±0.03749
Cz	1.42726±0.02372	1.40644±0.0302	1.45842±0.04433	1.43204±0.02829
C4	1.53373±0.03737	1.48363±0.02568	1.53135±0.03491	1.50151±0.02521
CP5	1.52806±0.02783	1.45567±0.01843	1.50595±0.03433	1.46293±0.02285
CP1	1.43617±0.02479	1.39018±0.01798	1.43724±0.02811	1.41518±0.0197
CPz	1.43135±0.02449	1.40103±0.0369	1.43886±0.03616	1.39447±0.02778
CP2	1.43823±0.02643	1.43018±0.02566	1.46871±0.03248	1.4049±0.02566
CP6	1.49987±0.03231	1.43913±0.0272	1.47677±0.03824	1.4151±0.02688
P7	1.52634±0.02712	1.44831±0.02553	1.50823±0.02414	1.4852±0.01947
P3	1.44631±0.03647	1.40561±0.02557	1.468±0.03367	1.40951±0.02743
Pz	1.41813±0.02558	1.35703±0.02442	1.38808±0.02559	1.3667±0.01779
P4	1.41023±0.03194	1.37535±0.02666	1.44128±0.04268	1.3352±0.02579
P8	1.46124±0.0255	1.42966±0.03891	1.46865±0.04276	1.40353±0.0271
O1	1.45884±0.02745	1.43364±0.03205	1.48166±0.03644	1.45174±0.02614
Poz	1.36628±0.03121	1.31811±0.02444	1.35782±0.02769	1.34153±0.02443
O2	1.46864±0.03001	1.45851±0.03344	1.49936±0.0297	1.43554±0.03162
T7	1.55774±0.0233	1.50202±0.02451	1.56484±0.02887	1.54434±0.02715
T8	1.51861±0.04054	1.48469±0.04315	1.51574±0.04728	1.46637±0.04219

Table S16. Quantitative analysis of Katz's fractal dimension of EEG across various electrodes in states of treatment group subjects. Mean±SEM.

Electrodes	No SD	SD	SD+Air-puff	SD+Nasal resp
Fp1	1.04472±0.00492	1.04327±0.00387	1.05426±0.00504	1.04766±0.00448
Fpz	1.04946±0.00787	1.04361±0.00448	1.05443±0.00716	1.04889±0.00643
Afz	1.04402±0.00449	1.04171±0.00426	1.05181±0.00684	1.04311±0.00541
Fp2	1.06062±0.00819	1.04833±0.00554	1.06297±0.00955	1.04956±0.00463
F7	1.06263±0.0103	1.04851±0.00312	1.06231±0.00567	1.05915±0.00445
F3	1.05367±0.0081	1.04675±0.00535	1.05746±0.00685	1.05462±0.00566
Fz	1.04066±0.00398	1.03913±0.00471	1.045±0.00572	1.04223±0.00546
F4	1.0457±0.00482	1.04208±0.00355	1.05679±0.0068	1.04465±0.00348
F8	1.06188±0.00927	1.0493±0.00326	1.06442±0.00608	1.05377±0.00602
FC5	1.05641±0.00571	1.0395±0.00455	1.05242±0.00672	1.04905±0.00854
FC1	1.04265±0.00608	1.03565±0.00433	1.04037±0.00477	1.04574±0.00586
FC2	1.04527±0.00534	1.04193±0.00553	1.05191±0.00525	1.04291±0.00472
FC6	1.05429±0.00924	1.03795±0.00354	1.05391±0.00578	1.04501±0.00655
C3	1.05478±0.00766	1.0374±0.00385	1.05104±0.00722	1.04251±0.00596
Cz	1.0344±0.00493	1.03387±0.00394	1.04213±0.00502	1.03842±0.00518
C4	1.05412±0.01084	1.03983±0.00481	1.05197±0.00757	1.04591±0.00723
CP5	1.05582±0.00991	1.04092±0.004	1.05304±0.00603	1.04473±0.00389
CP1	1.03259±0.0035	1.03118±0.00438	1.03673±0.00472	1.03554±0.00465
CPz	1.03627±0.00375	1.03698±0.00454	1.04367±0.00473	1.03798±0.00492
CP2	1.03381±0.00458	1.03429±0.00386	1.04052±0.00414	1.03445±0.00377
CP6	1.05411±0.0098	1.04605±0.00666	1.05512±0.00754	1.04262±0.00492
P7	1.07619±0.00797	1.05733±0.00573	1.06978±0.0067	1.0642±0.00442
P3	1.04669±0.00946	1.03886±0.00493	1.0483±0.00596	1.03871±0.00349
Pz	1.03788±0.00353	1.03457±0.00504	1.0414±0.00626	1.03774±0.00606
P4	1.04583±0.00487	1.04433±0.00619	1.06504±0.01237	1.04662±0.00669
P8	1.05962±0.00456	1.05591±0.00722	1.06586±0.00681	1.0557±0.00449
O1	1.06271±0.00439	1.06047±0.00539	1.0693±0.00667	1.06127±0.00532
Poz	1.04472±0.00428	1.04092±0.00434	1.05073±0.00629	1.04513±0.00606
O2	1.06416±0.00534	1.06315±0.00712	1.07157±0.00727	1.06105±0.00587
T7	1.08331±0.00999	1.05703±0.00702	1.07102±0.00734	1.06568±0.00646
T8	1.06001±0.01007	1.05597±0.00635	1.06566±0.00716	1.05525±0.00816

Table S17. Quantitative analysis of sample entropy of EEG across various electrodes in states of treatment group subjects. Mean±SEM.

Electrodes	No SD	SD	SD+Air-puff	SD+Nasal resp
Fp1	1.19396±0.13443	1.01782±0.10343	1.19639±0.12326	1.12533±0.10509
Fpz	1.03069±0.06693	0.99274±0.06475	1.10345±0.07087	0.93176±0.05592
AFz	0.98888±0.07444	0.90257±0.04853	1.07318±0.087	0.92484±0.05825
Fp2	1.21475±0.10468	1.03214±0.07932	1.15705±0.12307	1.06911±0.07547
F7	1.05495±0.04659	1.00555±0.04097	1.19192±0.07207	1.09296±0.05964
F3	1.06882±0.06377	1.08753±0.05827	1.14403±0.05315	1.16677±0.05541
Fz	0.94651±0.06789	0.88518±0.06062	0.99409±0.07239	0.93188±0.06951
F4	1.07277±0.07816	0.98634±0.04749	1.07572±0.05129	0.93912±0.05813
F8	1.21937±0.05438	1.07325±0.05382	1.22299±0.07843	1.17909±0.07891
FC5	1.21707±0.04824	1.10058±0.04771	1.30023±0.0798	1.17127±0.03799
FC1	1.11168±0.07875	1.01479±0.05391	1.05243±0.06462	1.12651±0.07878
FC2	1.05515±0.06617	0.89176±0.07132	1.05248±0.05449	0.92743±0.05499
FC6	1.2104±0.04403	1.12418±0.04333	1.22165±0.07158	1.07039±0.04963
C3	1.32416±0.06546	1.12817±0.05815	1.29542±0.05925	1.12765±0.06561
Cz	0.80977±0.06299	0.85626±0.04867	0.99138±0.05535	0.90565±0.06355
C4	1.21347±0.07771	1.19367±0.07648	1.30673±0.0714	1.20222±0.05755
CP5	1.23518±0.04152	1.13317±0.0535	1.29723±0.05631	1.12188±0.04165
CP1	1.02336±0.04366	0.94037±0.04515	1.03657±0.05905	0.98033±0.05524
CPz	1.02232±0.05106	0.91893±0.04784	1.01783±0.05764	0.9222±0.03988
CP2	1.04315±0.04555	0.97249±0.06564	1.05828±0.07819	0.93485±0.04054
CP6	1.14333±0.07429	1.08645±0.06597	1.18795±0.07371	1.03903±0.05944
P7	1.25678±0.04147	1.18272±0.05162	1.28087±0.08864	1.18052±0.05334
P3	1.11425±0.07205	1.02916±0.07277	1.15185±0.06152	0.96825±0.05029
Pz	0.94467±0.05004	0.86529±0.039	0.95169±0.05194	0.86676±0.04599
P4	1.01925±0.0714	0.86476±0.05411	1.04346±0.08404	0.81062±0.05172
P8	1.24627±0.07065	1.11477±0.08872	1.29563±0.08869	1.14516±0.08958
O1	1.10087±0.05692	1.11831±0.06461	1.23333±0.0888	1.11724±0.05859
POz	0.91272±0.05956	0.85312±0.04311	0.93161±0.05588	0.8515±0.0378
O2	1.05668±0.05155	1.09033±0.06608	1.19569±0.08042	1.06138±0.07286
T7	1.32666±0.05258	1.13774±0.05878	1.3663±0.06457	1.27587±0.05193
T8	1.13145±0.0892	1.17625±0.06929	1.25505±0.04722	1.0515±0.07341

Table S18. Results of ANOVA analysis of EEG delta power across various electrodes in treatment group subjects.

Electrodes	F(DFn,DFd), p value
**Fp1**	$F_{2.369,28.43}$=7.138, p=0.0020
**Fpz**	$F_{2.365,28.38}$=1.939, p=0.1564
**AFz**	$F_{1.501,18.01}$=1.155, p=0.3224
**Fp2**	$F_{2.055,24.66}$=4.024, p=0.0298
**F7**	$F_{2.315,27.78}$=3.633, p=0.0340
**F3**	$F_{1.048,12.58}$=1.485, p=0.2472
**Fz**	$F_{1.396,16.75}$=1.353, p=0.2751
**F4**	$F_{1.038,12.45}$=0.808, p=0.3904
**F8**	$F_{1.587,19.04}$=2.235, p=0.1420
**FC5**	$F_{1.734,20.81}$=3.112, p=0.0718
**FC1**	$F_{1.417,17.01}$=1.460, p=0.2543
**FC2**	$F_{1.649,19.79}$=2.266, p=0.1369
**FC6**	$F_{1.165,13.98}$=1.339, p=0.2740
**C3**	$F_{1.274,15.28}$=0.908, p=0.3803
**Cz**	$F_{1.844,22.13}$=2.165, p=0.1415
**C4**	$F_{1.620,19.44}$=2.232, p=0.1412
**CP5**	$F_{1.467,17.61}$=0.493, p=0.5621
**CP1**	$F_{2.272,27.27}$=2.872, p=0.0678
**CPz**	$F_{1.937,23.24}$=3.244, p=0.0586
**CP2**	$F_{2.183,26.19}$=3.572, p=0.0390
**CP6**	$F_{1.573,18.88}$=0.923, p=0.3926
**P7**	$F_{1.368,16.42}$=0.502, p=0.5452
**P3**	$F_{1.449,17.38}$=1.502, p=0.2463
**Pz**	$F_{2.128,25.53}$=2.760, p=0.0792
**P4**	$F_{1.269,15.23}$=1.149, p=0.3167
**P8**	$F_{1.881,22.58}$=0.624, p=0.5354
**O1**	$F_{1.515,18.18}$=2.011, p=0.1692
**POz**	$F_{1.269,15.23}$=1.149, p=0.3167
**O2**	$F_{1.319,15.83}$=1.898, p=0.1879
**T7**	$F_{1.936,23.23}$=2.052, p=0.1523
**T8**	$F_{1.286,15.43}$=1.414, p=0.2620
**Topoplot**	$F_{2.072,62.15}$=24.84, p<0.0001

Table S19. Results of ANOVA analysis of EEG theta power across various electrodes in treatment group subjects.

Electrodes	F(DFn,DFd), p value
**Fp1**	$F_{2.110,25.32}$=3.086, p=0.0608
**Fpz**	$F_{2.632,31.58}$=3.411, p=0.0343
**AFz**	$F_{1.765,21.17}$=2.854, p=0.0854
**Fp2**	$F_{2.601,31.21}$=5.355, p=0.0059
**F7**	$F_{2.471,29.66}$=7.472, p=0.0013
**F3**	$F_{2.421,29.05}$=3.648, p=0.0313
**Fz**	$F_{2.191,26.29}$=8.635, p=0.0010
**F4**	$F_{2.249,26.98}$=4.367, p=0.0194
**F8**	$F_{2.191,26.29}$=1.654, p=0.2102
**FC5**	$F_{1.902,22.83}$=2.250, p=0.1302
**FC1**	$F_{2.120,25.44}$=2.108, p=0.1369
**FC2**	$F_{1.629,19.55}$=0.117, p=0.8511
**FC6**	$F_{1.985,23.82}$=0.808, p=0.4567
**C3**	$F_{1.795,21.54}$=0.303, p=0.7179
**Cz**	$F_{1.630,19.56}$=1.292, p=0.2908
**C4**	$F_{2.364,28.36}$=1.607, p=0.2157
**CP5**	$F_{2.267,27.20}$=2.623, p=0.0848
**CP1**	$F_{2.115,25.38}$=2.629, p=0.0891
**CPz**	$F_{1.897,22.77}$=1.151, p=0.3315
**CP2**	$F_{1.546,18.55}$=1.678, p=0.2154
**CP6**	$F_{2.123,25.47}$=3.294, p=0.0508
**P7**	$F_{1.815,21.78}$=1.976, p=0.1655
**P3**	$F_{1.909,22.91}$=3.002, p=0.0716
**Pz**	$F_{1.576,18.91}$=1.065, p=0.3490
**P4**	$F_{1.603,19.24}$=2.063, p=0.1605
**P8**	$F_{1.804,21.65}$=2.415, p=0.1173
**O1**	$F_{2.719,32.63}$=2.681, p=0.0681
**POz**	$F_{1.654,19.85}$=2.923, p=0.0853
**O2**	$F_{1.957,23.49}$=1.804, p=0.1871
**T7**	$F_{2.073,24.87}$=3.594, p=0.0411
**T8**	$F_{1.588,19.05}$=0.875, p=0.4098
**Topoplot**	$F_{2.101,63.03}$=66.97, p<0.0001

Table S20. Results of ANOVA analysis of EEG alpha power across various electrodes in treatment group subjects.

Electrodes	F(DFn,DFd), p value
Fp1	$F_{1.984,23.81}$=1.298, p=0.2915
Fpz	$F_{1.956,23.47}$=1.676, p=0.2090
AFz	$F_{1.049,12.59}$=1.148, p=0.3076
Fp2	$F_{1.087,13.05}$=1.115, p=0.3166
F7	$F_{2.120,25.45}$=0.688, p=0.5199
F3	$F_{1.119,13.43}$=0.940, p=0.3606
Fz	$F_{1.039,12.47}$=1.095, p=0.3182
F4	$F_{1.181,14.17}$=1.190, p=0.3044
F8	$F_{1.410,16.93}$=0.547, p=0.5285
FC5	$F_{1.366,16.39}$=1.460, p=0.2541
FC1	$F_{1.054,12.64}$=0.979, p=0.3461
FC2	$F_{1.597,19.17}$=0.587, p=0.5290
FC6	$F_{1.483,17.80}$=0.852, p=0.4115
C3	$F_{1.989,23.87}$=1.076, p=0.3566
Cz	$F_{1.487,17.85}$=0.196, p=0.7592
C4	$F_{1.344,16.12}$=0.733, p=0.4438
CP5	$F_{1.156,13.87}$=1.726, p=0.2127
CP1	$F_{1.055,12.67}$=0.857, p=0.3781
CPz	$F_{1.518,18.22}$=0.645, p=0.4952
CP2	$F_{1.589,19.07}$=1.331, p=0.2816
CP6	$F_{1.451,17.42}$=0.596, p=0.3865
P7	$F_{1.562,18.75}$=0.243, p=0.7321
P3	$F_{1.316,15.79}$=1.001, p=0.3563
Pz	$F_{1.042,12.51}$=0.823, p=0.3044
P4	$F_{1.457,17.49}$=2.624, p=0.1128
P8	$F_{1.778,21.34}$=1.038, p=0.3636
O1	$F_{1.796,21.56}$=0.537, p=0.5734
POz	$F_{1.131,13.58}$=0.840, p=0.3898
O2	$F_{1.559,18.71}$=0.978, p=0.3745
T7	$F_{1.095,13.14}$=0.0887, p=0.7930
T8	$F_{1.198,14.37}$=2.028, p=0.1755
Topoplot	$F_{1.324,39.73}$=9.373, p=0.0019

Table S21. Results of ANOVA analysis of EEG beta power across various electrodes in treatment group subjects.

Electrodes	F(DFn,DFd), p value
Fp1	$F_{2.144,25.73}$=4.716, p=0.0163
Fpz	$F_{1.919,23.03}$=0.691, p=0.5053
AFz	$F_{1.680,20.16}$=0.469, p=0.5995
Fp2	$F_{1.825,21.90}$=1.580, p=0.2289
F7	$F_{2.411,28.94}$=5.217, p=0.0083
F3	$F_{1.429,17.15}$=1.636, p=0.2236
Fz	$F_{1.727,20.73}$=2.143, p=0.1475
F4	$F_{1.415,16.97}$=4.296, p=0.0422
F8	$F_{2.092,25.11}$=2.662, p=0.0873
FC5	$F_{1.906,22.88}$=1.820, p=0.1858
FC1	$F_{1.816,21.79}$=1.781, p=0.1939
FC2	$F_{1.573,18.88}$=3.770, p=0.0509
FC6	$F_{2.585,31.02}$=2.436, p=0.0912
C3	$F_{2.046,24.55}$=2.419, p=0.1089
Cz	$F_{1.779,21.35}$= 2.668, p=0.0975
C4	$F_{1.388,16.66}$=1.937, p=0.1815
CP5	$F_{1.599,19.19}$=2.463, p=0.1203
CP1	$F_{1.276,15.31}$=3.293, p=0.0815
CPz	$F_{2.081,24.97}$=3.220, p=0.0553
CP2	$F_{1.816,21.79}$=1.317, p=0.2862
CP6	$F_{1.915,22.98}$=2.568, p=0.1005
P7	$F_{2.212,26.55}$=4.882, p=0.0134
P3	$F_{1.269,15.23}$=1.801, p=0.2013
Pz	$F_{1.741,20.89}$=2.768, p=0.0919
P4	$F_{1.949,23.39}$=5.727, p=0.0099
P8	$F_{1.867,22.40}$=1.386, p=0.2697
O1	$F_{2.010,24.12}$=1.215, p=0.3143
POz	$F_{1.471,17.65}$=0.891, p=0.3979
O2	$F_{1.963,23.55}$=1.037, p=0.3691
T7	$F_{1.528,18.34}$=2.846, p=0.0948
T8	$F_{1.594,19.13}$=1.438, p=0.2587
Topoplot	$F_{2.509,75.26}$=35.15, p<0.0001

Table S22. Results of ANOVA analysis of EEG slow gamma power across various electrodes in treatment group subjects

Electrodes	F(DFn,DFd), p value
**Fp1**	$F_{1.101,13.21}$=1.037, p=0.3350
**Fpz**	$F_{2.010,24.11}$=1.018, p=0.3767
**AFz**	$F_{1.777,21.32}$=1.715, p=0.2054
**Fp2**	$F_{1.684,20.21}$=1.364, p=0.2746
**F7**	$F_{1.914,22.97}$=2.162, p=0.1396
**F3**	$F_{1.967,23.61}$=1.786, p=0.1899
**Fz**	$F_{1.150,13.80}$=2.469, p=0.1366
**F4**	$F_{1.304,15.65}$=2.805, p=0.1066
**F8**	$F_{1.925,23.11}$=1.886, p=0.1753
**FC5**	$F_{1.743,20.91}$=1.192, p=0.3176
**FC1**	$F_{2.352,28.23}$=1.839, p=0.1725
**FC2**	$F_{1.590,19.08}$=3.753, p=0.0508
**FC6**	$F_{2.110,25.32}$=3.452, p=0.0450
**C3**	$F_{1.428,17.13}$=1.814, p=0.1969
**Cz**	$F_{1.783,21.40}$=2.011, p=0.1618
**C4**	$F_{1.141,13.69}$=2.789, p=0.1146
**CP5**	$F_{1.382,16.59}$=1.646, p=0.2224
**CP1**	$F_{1.664,19.97}$=3.615, p=0.0529
**CPz**	$F_{2.062,24.75}$=4.347, p=0.0232
**CP2**	$F_{1.429,17.15}$=2.970, p=0.0914
**CP6**	$F_{1.352,16.22}$=2.993, p=0.0933
**P7**	$F_{2.100,25.20}$=5.461, p=0.0098
**P3**	$F_{1.053,12.63}$=1.442, p=0.2541
**Pz**	$F_{1.950,23.40}$=3.994, p=0.0331
**P4**	$F_{1.030,12.36}$=2.100, p=0.1723
**P8**	$F_{2.092,25.10}$=3.442, p=0.0459
**O1**	$F_{1.880,22.56}$=2.896, p=0.0787
**POz**	$F_{1.204,14.44}$=1.033, p=0.3420
**O2**	$F_{1.497,17.97}$=2.494, p=0.1215
**T7**	$F_{1.898,22.78}$=3.536, p=0.0481
**T8**	$F_{1.419,17.02}$=2.026, p=0.1701
**Topoplot**	$F_{2.365,70.96}$=31.89, p<0.0001

Table S23. Results of ANOVA analysis of EEG medium gamma power across various electrodes in treatment group subjects

Electrodes	F(DFn,DFd), p value
**Fp1**	$F_{1.157,13.88}$=1.140, p=0.3143
**Fpz**	$F_{1.868,22.41}$=1.903, p=0.1742
**AFz**	$F_{1.587,19.04}$=2.453, p=0.1215
**Fp2**	$F_{1.489,17.87}$=1.709, p=0.2112
**F7**	$F_{1.297,15.57}$=1.317, p=0.2809
**F3**	$F_{2.211,26.53}$=1.639, p=0.2117
**Fz**	$F_{1.057,12.69}$=2.179, p=0.1641
**F4**	$F_{2.005,24.06}$=4.992, p=0.0153
**F8**	$F_{1.813,21.75}$=1.805, p=0.1902
**FC5**	$F_{1.381,16.57}$=0.595, p=0.5031
**FC1**	$F_{2.013,24.15}$=3.763, p=0.0375
**FC2**	$F_{1.697,20.36}$=5.185, p=0.0189
**FC6**	$F_{1.430,17.16}$=1.591, p=0.2309
**C3**	$F_{1.430,17.16}$=1.591, p=0.2309
**Cz**	$F_{1.825,21.90}$=3.138, p=0.0673
**C4**	$F_{1.307,15.68}$=2.549, p=0.1244
**CP5**	$F_{1.246,14.96}$=1.672, p=0.2195
**CP1**	$F_{1.416,16.99}$=2.975, p=0.0917
**CPz**	$F_{1.858,22.30}$=5.545, p=0.0125
**CP2**	$F_{1.245,14.94}$=2.859, p=0.1058
**CP6**	$F_{1.711,20.53}$=2.127, p=0.1499
**P7**	$F_{2.267,27.20}$=5.935, p=0.0056
**P3**	$F_{1.685,20.22}$=5.866, p=0.0128
**Pz**	$F_{2.000,24.00}$=9.157, p=0.0011
**P4**	$F_{2.111,25.34}$=5.271, p=0.0111
**P8**	$F_{2.233,26.80}$=4.433, p=0.0187
**O1**	$F_{1.565,18.78}$=1.116, p=0.3343
**POz**	$F_{2.080,24.96}$=3.810, p=0.0345
**O2**	$F_{1.857,22.28}$=3.516, p=0.0501
**T7**	$F_{1.563,18.76}$=1.567, p=0.2341
**T8**	$F_{1.516,18.20}$=0.968, p=0.3755
**Topoplot**	$F_{2.174,65.22}$=32.68, p<0.0001

Table S24. Results of ANOVA analysis of EEG fast gamma power across various electrodes in treatment group subjects

Electrodes	F(DFn,DFd), p value
**Fp1**	$F_{1.180,14.17}$=1.214, p=0.2992
**Fpz**	$F_{1.796,21.55}$=1.953, p=0.1691
**AFz**	$F_{1.274,15.29}$=1.862, p=0.1933
**Fp2**	$F_{1.316,15.79}$=1.661, p=0.2207
**F7**	$F_{1.061,12.73}$=0.970, p=0.3487
**F3**	$F_{1.528,18.34}$=1.589, p=0.2305
**Fz**	$F_{1.024,12.29}$=1.479, p=0.2478
**F4**	$F_{1.851,22.21}$=3.294, p=0.0592
**F8**	$F_{1.232,14.78}$=1.470, p=0.2516
**FC5**	$F_{1.383,15.59}$=0.628, p=0.4893
**FC1**	$F_{2.123,25.47}$=2.866, p=0.0726
**FC2**	$F_{2.064,24.76}$=4.287, p=0.0242
**FC6**	$F_{1.454,17.44}$=2.443, p=0.1273
**C3**	$F_{1.445,17.33}$=1.387, p=0.2685
**Cz**	$F_{1.524,18.29}$=1.991, p=0.1714
**C4**	$F_{1.343,16.12}$=2.411, p=0.1341
**CP5**	$F_{1.208,14.50}$=1.175, p=0.3086
**CP1**	$F_{1.206,14.47}$=2.356, p=0.1437
**CPz**	$F_{1.440,17.29}$=2.901, p=0.0950
**CP2**	$F_{1.150,13.80}$=2.582, p=0.1281
**CP6**	$F_{1.054,12.65}$=1.370, p=0.2660
**P7**	$F_{2.276,27.31}$=4.198, p=0.0217
**P3**	$F_{1.960,23.52}$=5.530, p=0.0111
**Pz**	$F_{1.772,21.27}$=8.763, p=0.0023
**P4**	$F_{1.118,13.41}$=0.8907, p=0.3739
**P8**	$F_{2.432,29.19}$=2.702, p=0.0742
**O1**	$F_{1.750,21.00}$=2.074, p=0.1549
**POz**	$F_{1.165,13.98}$=1.127, p=0.3175
**O2**	$F_{2.001,24.02}$=3.439, p=0.0486
**T7**	$F_{1.417,17.00}$=2.086, p=0.1633
**T8**	$F_{1.595,19.14}$=1.005, p=0.3676
**Topoplot**	$F_{2.087,62.60}$=36.33, p<0.0001

Table S25. Results of ANOVA analysis of Higuchi's fractal dimension of EEG across various electrodes in treatment group subjects

Electrodes	F(DFn,DFd), p value
**Fp1**	$F_{1.973,23.67}$=2.007, p=0.1571
**Fpz**	$F_{2.439,29.27}$=2.933, p=0.0596
**AFz**	$F_{1.924,23.09}$=2.871, p=0.0788
**Fp2**	$F_{2.625,31.50}$=4.535, p=0.0120
**F7**	$F_{1.645,19.74}$=1.001, p=0.3708
**F3**	$F_{1.810,21.72}$=1.435, p=0.2587
**Fz**	$F_{1.533,18.40}$=2.697, p=0.1046
**F4**	$F_{2.020,24.24}$=2.656, p=0.0901
**F8**	$F_{2.019,24.22}$=3.172, p=0.0593
**FC5**	$F_{2.377,28.53}$=4.041, P=0.0229
**FC1**	$F_{1.759,21.10}$=1.800, p=0.1921
**FC2**	$F_{2.312,27.75}$=3.624, p=0.0344
**FC6**	$F_{1.757,21.08}$=3.774, p=0.0446
**C3**	$F_{2.035,24.42}$=3.916, p=0.0329
**Cz**	$F_{1.896,22.75}$=0.810, p=0.4513
**C4**	$F_{1.768,21.22}$=1.787, p=0.1941
**CP5**	$F_{2.529,30.35}$=4.450, p=0.0141
**CP1**	$F_{2.041,24.49}$=2.329, p=0.1177
**CPz**	$F_{1.517,18.21}$=1.990, p=0.1718
**CP2**	$F_{2.125,25.50}$=1.466, p=0.2497
**CP6**	$F_{1.802,21.62}$=4.036, p=0.0360
**P7**	$F_{2.011,24.14}$=4.902, p=0.0162
**P3**	$F_{1.865,22.38}$=3.126, p=0.0665
**Pz**	$F_{1.827,21.92}$=3.861, p=0.0399
**P4**	$F_{1.754,21.05}$=5.473, p=0.0148
**P8**	$F_{1.775,21.30}$=2.781, p=0.0898
**O1**	$F_{2.193,26.31}$=1.622, P=0.2153
**POz**	$F_{1.363,16.35}$=2.028, p=0.1714
**O2**	$F_{2.128,25.53}$=3.700, p=0.0365
**T7**	$F_{2.187,26.25}$=2.333, p=0.1127
**T8**	$F_{2.370,28.45}$=1.987, p=0.1491
**Topoplot**	$F_{2.229,66.88}$=98.94, p<0.0001

Table S26. Results of ANOVA analysis of Katz's fractal dimension of EEG across various electrodes in treatment group subjects

Electrodes	F(DFn,DFd), p value
**Fp1**	$F_{2.281,27.36}$=4.462, p=0.0174
**Fpz**	$F_{2.158,25.90}$=2.040, p=0.1476
**AFz**	$F_{1944,23.33}$=2.307, p=0.1230
**Fp2**	$F_{2.076,24.91}$=1.911, p=0.1679
**F7**	$F_{1.468,17.61}$=1.447, p=0.2568
**F3**	$F_{2.221,26.65}$=1.271, p=0.2994
**Fz**	$F_{1.505,18.06}$=1.262, p=0.2962
**F4**	$F_{1.942,23.31}$=3.537, p=0.0467
**F8**	$F_{1.939,23.27}$=2.482, p=0.1067
**FC5**	$F_{2.482,29.79}$=3.377, p=0.0386
**FC1**	$F_{1.844,22.13}$=1.667, p=0.2126
**FC2**	$F_{1.806,21.67}$=4.265, p=0.0307
**FC6**	$F_{1.922,23.06}$=2.958, p=0.0737
**C3**	$F_{1.480,17.76}$=2.580, p=0.1152
**Cz**	$F_{2.045,24.54}$=1.416, p=0.2621
**C4**	$F_{1.271,15.25}$=1.770, p=0.2054
**CP5**	$F_{1.864,22.37}$=2.686, p=0.0932
**CP1**	$F_{2.498,29.98}$=3.791, p=0.0261
**CPz**	$F_{2.177,26.13}$=2.594, p=0.0898
**CP2**	$F_{1.752,21.03}$=2.943, p=0.0804
**CP6**	$F_{1.468,17.61}$=3.294, p=0.0732
**P7**	$F_{1.943,23.32}$=4.265, p=0.0273
**P3**	$F_{1.330,15.96}$=2.885, p=0.1006
**Pz**	$F_{1.650,19.80}$=1.915, p=0.1781
**P4**	$F_{1.359,16.31}$=5.665, p=0.0219
**P8**	$F_{1.846,22.15}$=2.853, p=0.0826
**O1**	$F_{2.112,25.35}$=1.180, p=0.3256
**POz**	$F_{1.696,20.35}$=2.925, p=0.0836
**O2**	$F_{1.993,23.91}$=2.005, p=0.1568
**T7**	$F_{2.526,30.32}$=4.436, p=0.0143
**T8**	$F_{2.345,28.14}$=1.209, p=0.3185
**Topoplot**	$F_{1.943,58.28}$=50.36, p<0.0001

Table S27. Results of ANOVA analysis of sample entropy of EEG across various electrodes in treatment group subjects

Electrodes	$F_{(DFn,DFd)}$, p value
**Fp1**	$F_{2.676,32.11}$=2.015, p=0.1372
**Fpz**	$F_{2.352,28.22}$=3.054, p=0.0555
**AFz**	$F_{2.187,26.25}$=3.098, p=0.0579
**Fp2**	$F_{2.414,28.97}$=1.913, p=0.1591
**F7**	$F_{2.171,26.06}$=4.798, p=0.0149
**F3**	$F_{2.422,27.53}$=1.689, p=0.1978
**Fz**	$F_{2.294,22.72}$=1.931, p=0.1593
**F4**	$F_{2.012,24.14}$=3.135, p=0.0613
**F8**	$F_{2.462,29.55}$=3.120, p=0.0495
**FC5**	$F_{2.050,24.60}$=3.797, p=0.0356
**FC1**	$F_{2.029,24.35}$=1.555, p=0.2313
**FC2**	$F_{2.623,31.47}$=2.826, p=0.0610
**FC6**	$F_{1.637,19.64}$=3.477, p=0.0590
**C3**	$F_{2.054,24.64}$=4.396, p=0.0225
**Cz**	$F_{2.136,25.63}$=2.087, p=0.1421
**C4**	$F_{1.453,17.44}$=1.463, p=0.2538
**CP5**	$F_{2.446,29.35}$=4.051, p=0.0215
**CP1**	$F_{2.469,29.63}$=2.616, p=0.0795
**CPz**	$F_{1.746,20.95}$=3.836, p=0.0431
**CP2**	$F_{2.283,27.39}$=1.692, p=0.2000
**CP6**	$F_{1.663,19.95}$=2.220, p=0.1412
**P7**	$F_{2.190,26.28}$=1.323, p=0.2851
**P3**	$F_{2.154,25.85}$=3.519, p=0.0415
**Pz**	$F_{1.929,23.15}$=2.228, p=0.1318
**P4**	$F_{2.199,26.39}$=4.589, p=0.0170
**P8**	$F_{1.893,22.72}$=3.573, p=0.0469
**O1**	$F_{2.164,25.97}$=2.415, p=0.1055
**POz**	$F_{1.424,17.09}$=1.556, p=0.2370
**O2**	$F_{2.151,25.82}$=3.710, p=0.0356
**T7**	$F_{2.589,31.07}$=3.643, p=0.0282
**T8**	$F_{2.213,26.56}$=4.076, p=0.0253
**Topoplot**	$F_{2.587,77.61}$=56.12, p<0.0001

Table S28. Specific p-values for statistical comparisons of intra-DMN cross-correlation among different states and No SD across various frequency bands. An increase in correlation is indicated by a plus sign, while a decrease is indicated by a minus sign. L (left), Cnt (center) and R (right). vmPFC: ventromedial prefrontal cortex, dmPFC: dorsomedial prefrontal cortex, PCC: posterior cingulate cortex, SD: sleep deprivation, SD+AP: sleep deprivation+nasal air-puffs, SD+NR: sleep deprivation+nasal respiration; paired t-test.

Frequency band	DMN paired region	SD vs. No SD	SD+AP vs. No SD	SD+NR vs. No SD
Delta	dmPFC(Cnt)-dmPFC(R)	-0.0102	-0.4854	0.9084
	dmPFC(L)-dmPFC(R)	0.9294	-0.0049	-0.0445
	Parietal(L)-Parietal(R)	0.4692	-0.0051	-0.2052
	dmPFC(L)-Parietal(R)	0.7866	-0.1019	0.0015
Theta	dmPFC(Cnt)-dmPFC(R)	-0.0261	-0.3518	0.4504
	dmPFC(Cnt)-PCC(R)	-0.0414	-0.1380	-0.3638
	dmPFC(Cnt)-PCC(L)	0.9878	0.0290	0.1509
	dmPFC(Cnt)-Preconeus (R)	0.6243	-0.0127	0.4903
	vmPFC(R)-dmPFC(R)	-0.5232	-0.6594	0.0223
	Preconeus(L)-Preconeus(R)	0.4292	0.2038	0.0219
Alpha	Preconeus(L)-Preconeus(R)	0.0132	0.0136	0.0106
Beta	Preconeus(L)-Preconeus(R)	0.0219	0.0074	0.0406
	Preconeus(L)-Parietal(R)	0.0177	0.7074	0.0120
	Preconeus(Cnt)-vmPFC(R)	0.3482	0.3561	-0.0174
	PCC(Cnt)-Preconeus(L)	0.2269	0.8009	-0.0392
	Preconeus(R)-Parietal(R)	0.1122	-0.9788	0.04023
	PCC(Cnt)-Preconeus(R)	0.2195	0.7067	-0.0030
	PCC(Cnt)-Parietal(R)	0.5012	0.8274	-0.0268
Slow gamma	PCC(Cnt)-Parietal(R)	-0.0143	0.1279	-0.0268
	PCC(Cnt)-dmPFC(R)	0.0086	0.3825	0.1717
	dmPFC(Cnt)-PCC(Cnt)	0.3869	0.6668	0.0124
	PCC(Cnt)-Preconeus(L)	0.2991	0.5519	-0.0245
	PCC(Cnt)-Preconeus(R)	0.2826	0.3038	-0.0282
	PCC(R)-Preconeus(R)	0.3617	0.5349	0.0497
Medium gamma	PCC(Cnt)-Parietal(R)	-0.0493	0.1252	0.1679
	dmPFC(Cnt)-Preconeus(Cnt)	0.0425	-0.7708	-0.1840
	dmPFC(Cnt)-dmPFC(L)	-0.7821	-0.7343	-0.0065
Fast gamma	PCC(Cnt)-Preconeus(R)	-0.0391	0.2027	-0.0165
	dmPFC(Cnt)-dmPFC(L)	-0.5835	-0.1283	-0.0139
	Preconeus(Cnt)-vmPFC(L)	0.0485	0.2797	0.7573
	Preconeus(Cnt)-vmPFC(R)	0.0131	0.5812	0.6784
	PCC(Cnt)-Preconeus(R)	-0.0453	0.70267	-0.0365
	Preconeus(Cnt)-PCC(L)	0.2606	-0.7600	-0.0404
	PCC(Cnt)-Parietal(R)	0.3439	-0.0124	0.3759

Table S29. Specific p-values for statistical comparisons of intra-DMN coherence among different states and No SD across various frequency bands. An increase in coherence is indicated by a plus sign, while a decrease is indicated by a minus sign. L (left), Cnt (center) and R (right). vmPFC: ventromedial prefrontal cortex, dmPFC: dorsomedial prefrontal cortex, PCC: posterior cingulate cortex, SD: sleep deprivation, SD+AP: sleep deprivation+nasal air-puffs, SD+NR: sleep deprivation+nasal respiration; paired t-test.

Frequency band	DMN paired region	SD vs. No SD	SD+AP vs. No SD	SD+NR vs. No SD
Delta	dmPFC(Cnt)-dmPFC(R)	-0.0292	-0.4009	0.6933
	vmPFC(R)-dmPFC(R)	-0.0240	-0.1193	0.8584
	PCC(L)-PCC(R)	0.0221	0.0904	0.4116
	vmPFC(L)-Parietal(L)	-0.1546	-0.0389	0.7976
	dmPFC(L)-Parietal(L)	-0.5668	-0.0393	-0.8679
	Parietal(L)- Parietal(R)	-0.2780	-0.0251	0.8057
	Parietal(L)-Precuneus(Cnt)	0.8533	-0.0336	0.5623
	dmPFC(Cnt)-Precuneus(R)	0.7977	0.0376	0.3659
	vmPFC(R)-PCC(R)	0.7229	-0.9839	0.0201
Theta	vmPFC(L)-Precuneus(Cnt)	0.1321	0.0256	0.2769
	dmPFC(Cnt)-Precuneus(R)	0.6586	0.0084	0.0882
	Parietal(L)-Precuneus(R)	0.2735	0.0393	0.9692
	Precuneus(Cnt)-Precuneus(L)	0.4989	0.0379	0.7344
	PCC(L)-Precuneus(L)	0.4403	0.0386	0.3985
	dmPFC(R)-PCC(L)	-0.3384	-0.0329	-0.1422
	vmPFC(R)-dmPFC(R)	-0.9520	0.8039	0.0239
Alpha	PCC(Cnt)-Precuneus(L)	0.0133	0.5033	0.0215
	PCC(Cnt)-Precuneus(R)	0.0242	0.3386	0.0536
	vmPFC(R)-dmPFC(L)	0.1668	0.0108	0.1482
	dmPFC(Cnt)-Precuneus(R)	0.2567	0.0313	0.1057
	Parietal(L)-Precuneus(R)	0.3419	0.0281	0.1166
	dmPFC(R)-PCC(R)	-0.1780	-0.0243	-0.1967
	PCC(R)-Precuneus(L)	-0.0937	-0.0250	-0.0082
Beta	PCC(Cnt)-Precuneus(R)	0.0237	0.4537	0.0094
	PCC(L)-Precuneus(L)	-0.0331	-0.1378	-0.8681
	PCC(Cnt)-Precuneus(L)	0.1299	0.8569	0.0084
Slow gamma	PCC(Cnt)-Precuneus(R)	0.0363	0.4998	0.0276
	dmPFC(Cnt)-PCC(L)	-0.2092	-0.0039	-0.5861
	vmPFC(R)-Precuneus(R)	0.3670	0.5087	0.0379
Medium gamma	dmPFC(Cnt)-Parietal(R)	0.0253	0.0598	0.2164
	Parietal(R)-Precuneus(L)	0.0299	0.8844	0.2363
	PCC(Cnt)-Precuneus(R)	0.1478	0.3699	0.0406
